# Temperature Driven Changes in Benthic Bacterial Diversity Influences Biogeochemical Cycling in Coastal Sediments

**DOI:** 10.3389/fmicb.2018.01730

**Published:** 2018-08-22

**Authors:** Natalie Hicks, Xuan Liu, Richard Gregory, John Kenny, Anita Lucaci, Luca Lenzi, David M. Paterson, Katherine R. Duncan

**Affiliations:** ^1^The Scottish Association for Marine Science, Scottish Marine Institute, Oban, United Kingdom; ^2^Centre for Genomic Research, Institute of Integrative Biology, University of Liverpool, Liverpool, United Kingdom; ^3^Sediment Ecology Research Group, School of Biology, Scottish Oceans Institute, University of St Andrews, Fife, United Kingdom; ^4^Strathclyde Institute of Pharmacy and Biomedical Sciences, University of Strathclyde, Glasgow, United Kingdom

**Keywords:** benthic biogeochemistry, microbial communities, biogeochemical cycles, environmental change, benthic microbial ecology, marine sediments

## Abstract

Marine sediments are important sites for global biogeochemical cycling, mediated by macrofauna and microalgae. However, it is the microorganisms that drive these key processes. There is strong evidence that coastal benthic habitats will be affected by changing environmental variables (rising temperature, elevated CO_2_), and research has generally focused on the impact on macrofaunal biodiversity and ecosystem services. Despite their importance, there is less understanding of how microbial community assemblages will respond to environmental changes. In this study, a manipulative mesocosm experiment was employed, using next-generation sequencing to assess changes in microbial communities under future environmental change scenarios. Illumina sequencing generated over 11 million 16S rRNA gene sequences (using a primer set biased toward bacteria) and revealed Bacteroidetes and Proteobacteria dominated the total bacterial community of sediment samples. In this study, the sequencing coverage and depth revealed clear changes in species abundance within some phyla. Bacterial community composition was correlated with simulated environmental conditions, and species level community composition was significantly influenced by the mean temperature of the environmental regime (*p* = 0.002), but not by variation in CO_2_ or diurnal temperature variation. Species level changes with increasing mean temperature corresponded with changes in NH_4_ concentration, suggesting there is no functional redundancy in microbial communities for nitrogen cycling. Marine coastal biogeochemical cycling under future environmental conditions is likely to be driven by changes in nutrient availability as a direct result of microbial activity.

## Introduction

Marine sediments play a vital role in global biogeochemical cycling, particularly in terms of carbon, nitrogen and oxygen dynamics (Glud, 2008). The predicted global climate change scenarios (IPCC, 2014) will result in marine sediments being subjected to many environmental pressures, e.g., increasing mean temperature, greater temperature fluctuation, and increasing CO_2_ levels (ocean acidification: OA) (Doney et al., 2009; Dossena et al., 2012). As a direct consequence of rising atmospheric carbon emissions, global average temperature is expected to rise by ~4°C by 2100; and ocean pH, as a result of acidification, is predicted to decline to 7.8 in the same time period (0.2 pH units lower than pre-industrial levels) (Caldeira and Wickett, 2003; Kroeker et al., 2013; IPCC, 2014). It is recognized that many of the key ecosystem services (Beaumont et al., 2007) provided by marine benthic habitats are driven by microbial activity (Prosser and Head, 2007; Bertics and Ziebis, 2009; Gilbertson et al., 2012), such as the nitrogen fixation carried out by the cyanobacteria genera *Trichodesmium* and *Crocosphaera* (Hutchins et al., 2013).

Biogeochemical cycling within sediments, and at the sediment water interface, varies with sediment type (Aldridge et al., 2017; Hicks et al., 2017a), and this is reflected in the different microbial communities (Currie et al., 2017; Kitidis et al., 2017). Cohesive coastal sediments, such as those found in estuaries and intertidal mudflats, tend to have a high organic carbon content, and the sediment biogeochemical cycling is heavily influenced by diffusive processes (Hicks et al., 2017a). Considering the contribution of benthic microbes to ecosystem services (Bell et al., 2005), particularly biogeochemical cycling (Dyksma et al., 2016), it is vital that we understand how microbial population dynamics are likely to shift under future climate change scenarios, and how this may affect ecosystem service provision.

Climate driven changes, such as warming and elevated CO_2_, are known to alter many biogeochemical cycles, such as the nitrogen cycle (nitrification and ammonia oxidation) (Kitidis et al., 2011, 2017) which are mediated by microbial assemblages (Hutchins and Fu, 2017). There is substantial evidence that benthic systems will respond to predicted changes in temperature and CO_2_; both on an ecosystem and individual species level (Bulling et al., 2010; Hicks et al., 2011; Godbold and Solan, 2013; Cartaxana et al., 2015). Individual stressor studies have shown how warming elicits varied responses in microbial communities, with some heterotrophic bacteria responding positively with increasing growth (Vázquez-Domínguez et al., 2012), and other smaller bacteria decreasing in size (Moran et al., 2015), with implications for nutrient cycling in coastal sediments (Alsterberg et al., 2011). Changes in pH through ocean acidification (elevated CO_2_) also show mixed effects on benthic microbial communities, with abundance of ammonia oxidizing bacteria (AOB) and denitrifiers decreasing in Arctic sediments as a response to OA (Tait et al., 2013), although ammonia oxidization rates appeared unaffected (Kitidis et al., 2011).

Anthropogenically-driven environmental changes are likely to occur simultaneously, and integration of multiple stressors into experimental designs is likely to produce differing responses to those measured for single stressor studies (Crain et al., 2008; Kenworthy et al., 2016; Pendleton et al., 2016). This, combined with the natural variability in many intertidal systems (such as changes in temperature, salinity, exposure) (Benedetti-Cecchi et al., 2006; Molinos and Donohue, 2010; García Molinos and Donohue, 2011) adds to the complexity in interpreting and understanding stressor specific responses and potential shifts in microbial community composition (Fu et al., 2007).

The high diversity typically found within benthic microbial communities may make benthic ecosystems more resistant to environmental change (Kerfahi et al., 2014), ensuring the biogeochemical functions of microbial assemblages remain constant. Previous studies examining benthic microbial community composition and diversity have used a range of “fingerprinting” techniques, such as phospholipid fatty acid (PLFA) analysis to estimate biomass and identify key biomarkers (Findlay and Watling, 1998; Mayor et al., 2012; Sweetman et al., 2014; Main et al., 2015); terminal restriction fragment length polymorphism (T-RFLP) (Moss et al., 2006; Febria et al., 2012; Tait et al., 2015), and denaturing gradient gel electrophoresis (DGGE) (Bolhuis et al., 2013).

To-date, few studies have examined the effects of combined environmental stressors on microbial benthic communities (Currie et al., 2017), and to our knowledge this is the first to integrate natural variability as an additional stressor. This study uses next generation sequencing (Ilumina MiSeq) to identify changes in microbial community composition from a manipulative mesocosm study with a focus on biodiversity driven changes in biogeochemical function. Experimental environmental change variables included ambient and elevated CO_2_; elevated temperature; and temporal variability (diurnal temperature fluctuation) which is reflective of *in situ* changes in intertidal habitats. Predictions of future temperature elevation are often referred to as a mean global rise, and the diurnal variability of temperature in this experimental design represents the change in both mean temperature, but also the extremes experienced particularly in coastal and tidal ecosystems. The 16S rRNA gene was sequenced from environmental DNA extracted from the incubated intertidal cohesive sediment samples at the end of the experiment. This provides insight into microbial responses toward environmental change, and we discuss the implications on marine biogeochemical cycling. This study harnesses advanced sequencing technology to provide essential understanding of the global consequences of climate change on microbial community composition. We hypothesize that shifts in microbial community assemblages will be a response to changing environmental conditions, and this may be synergistic or additive.

## Materials and methods

### Sample collections and processing

Surface sediment (< 2 cm depth) was collected from the Ythan Estuary tidal mud flats in, Aberdeenshire, Scotland, UK (57° 20.085′N, 02° 0.206′ W) in spring/early summer 2008 and sieved (500 μm) in a seawater bath (UV sterilized, 10 μm filtered, salinity 33) to remove macrofauna. The sediment was left to settle for 48 h before the supernatant was removed. Additional microphytobenthos (MPB)-rich sediment was collected at the same time, and placed under constant light in a shallow tray for 48 h. The sediment was then homogenized and placed in perspex mesocosms to a depth of 10 cm (785 cm^3^), as previously described (Bulling et al., 2010; Hicks et al., 2011). The MPB-rich sediment was also homogenized and distributed (125 cm^3^) on the surface of sediment in each mesocosm, and topped up with seawater to an overlying depth of 20 cm. This water was replaced after 24 h to reduce any biogeochemical fluxes associated with sediment homogeneity and mesocosm assembly (Ieno et al., 2006). The mesocosms were then placed into environmental chambers for the duration of the experiment.

### Mesocosm experiments

Mesocosms were placed in two environmental chambers (V 4100, Vötsch Industrietechnik, temperature control ± 0.1°C), with each chamber running at one of two CO_2_ treatments [380 ppmv (ambient; L) and 1,000 ppmv (elevated; H)]. The experiments were run on a 12 h light-12 h dark (L/D) cycle using high intensity discharge sodium lamps (model GE11678, 400w × 2, average 300 μmoles m^−2^ s^−1^) to allow MPB photosynthesis. Eighteen unique environmental regimes were used, consisting of three mean temperatures (6°, 12°, and 18°C), two atmospheric CO_2_ concentrations, and three fluctuating temperature regimes (FTR = 1°, 3°, and 6°C) around the mean (one complete fluctuation cycle every 24 h). Experimental design included three replicates (*n* = 3) per environmental regime (Table 1, Figure S1).

**Table 1:** Manipulative mesocosm design, representing the sample ID and experimental conditions of all 18 unique treatments.

**Treatment ID**	**CO_2_ Treatment (ppmv)**	**Mean Temperature (^°^C)**	**Temperature Fluctuation (^°^C)**
L6-1	380	6	1
H6-3	1,000	6	3
L6-6	380	6	6
H6-1	1,000	6	1
L6-3	380	6	3
H6-6	1,000	6	6
H12-3	1,000	12	3
L12-1	380	12	1
H12-1	1,000	12	1
L12-6	380	12	6
L12-3	380	12	3
H12-6	1,000	12	6
L18-1	380	18	1
H18-3	1,000	18	3
H18-6	1,000	18	6
L18-3	380	18	3
H18-1	1,000	18	1
L18-6	380	18	6

Atmospheric CO_2_ concentrations were maintained as previously described (Bulling et al., 2010; Hicks et al., 2011). Mesocosms were randomly positioned within each environmental chamber to factor out any spatial heterogeneity effects. Each experiment was run for 7 days.

### MPB biomass and sediment sampling

MPB biomass was measured in each mesocosm prior to sediment and water sampling, using non-invasive Pulse Amplitude Modulated (PAM) fluorometry to estimate chlorophyll content, following the methodology described in Consalvey et al. (2005) and Hicks et al. (2011). Sediment samples were taken at the end of each experiment use the cryolander and contact core technique (Honeywill et al., 2002) using LN_2_ to freeze the sediment surface (diameter 50 mm, depth ~2–3 mm). Sediment samples were individually wrapped in foil and immediately stored in a −80°C freezer until DNA extraction.

### Nutrient analysis

Water samples (filtered at 0.45 μm) were taken from the overlying water in each mesocosm at the end of the experiment. NH_4_, NO_x_, (nitrate and nitrite) and PO_4_ concentration were measured using a flow through injector analyser (FIA Star 5010 series) with an artificial seawater carrier solution (Bulling et al., 2010).

### Isolation of sediment metagenomic DNA

Previously established protocols were used to extract high quality environmental DNA from all 54 sediment samples (Duncan et al., 2014, 2015). No blank DNA control was included in the experimental design; therefore, laboratory contamination cannot be ruled out (Salter et al., 2014). Thawed sediment was centrifuged to remove associated water and eDNA was extracted from approximately 200 mg of each sediment sample using the Fast DNA Spin Kit for Soils according to the manufacturer's recommendation (MP Biomedicals, Solon, OH, USA) and stored at −20°C. Concentration and integrity of isolated DNA was determined by UV spectroscopy and agarose gel electrophoresis (1% agarose, 1 × Tris-acetate-EDTA buffer, strained with ethidium bromide) (Sambrook et al., 1989). A total of 5 μL of extracted genomic DNA for each of the 54 samples was pipetted into a 96 well plate, after being diluted to 1 ng/μL and sent on dry ice overnight to “The Centre of Genomic Research,” Liverpool for sequencing. Samples from each treatment (*n* = 3) were named according to their environmental treatment e.g., H6-1 represents High CO2; 6°C mean temperature; and 1°C temperature fluctuation (Table 1).

### 16S rRNA gene amplification and sequencing

Environmental DNA was extracted from all 54 sediment samples and the 16S rRNA gene was amplified using primers 515F and 806R targeting the V4 region of the 16S rRNA gene, and thus biased to the amplification of bacterial DNA (Caporaso et al., 2011; D'Amore et al., 2016) and sequenced using an Illumina MiSeq platform. The read counts before and after adapter trimming and quality control are summarized (Table S1). Further analysis used only R1 and R2 reads and the samples H18-3b and L18-6c were excluded from the dataset due to low pair reads [ < 100 base pairs (bp)]. Following adaptor sequence removal and quality trimming, the remaining 52 samples contained between 149,199 and 1,107,840 trimmed reads (Table S1). Amplicon generation targeting the 16S rRNA gene was performed for each of the 54 environmental DNA samples, and amplified by 10 cycles of PCR using the Kapa enzyme (see Supplementary Material for detailed methodology). DNA concentrations were recorded using a Qubit fluorometer (ThermoFisher) and scanned on the Fragment Analyser (Advanced Analytical). This allowed pooling of samples based on a size selection of 350–650 base pairs. Sequencing was carried out on an Illumina MiSeq at 2 × 250 base pair (bp) paired-end sequencing with v2 chemistry. Fragmented PhiX phage was added to the sequence library in order to increase the sequence complexity. Sequences are published in the European Nucleotide Archive (ENA) under the study accession number PRJEB13670 and sample accession numbers ERS1124371-ERS1124422).

### Grouping sequences into operational taxonomic units

A metadata file was created to describe each sample, and an error calculation was run by clustering sequences at 99%, identifying and generating a consensus sequence for the cluster. Chimera detection used a dataset of 16S rRNA genes as potential “parent” sequences in addition to using the most common sequences in the dataset. Post-processing of the Illumina sequence reads included quality control and clustering reads into operational taxonomic units (OTUs) at 99% sequence identity. A minimum cluster size was set to remove clusters containing fewer than four sequences. OTU-picking was done using QIIME to cluster sequences, remove chimeras and define OTU abundance. This final dataset was then clustered at 97% sequence similarity to identify taxonomy from the Greengenes database, version 12.8 (McDonald et al., 2012), using the RPD classifier (Wang et al., 2007).

For detailed methodology on sequence procedures, including scripts used in QIIME, alpha and beta diversity and rarefaction statistical analysis, please see Supplementary Material.

### Metagenomic analysis

Over 11 million sequences (11,745,334) passed the quality control filters and all 52 samples were pooled into a single metadata file, which was processed for metagenomic analysis using QIIME (Caporaso et al., 2010). In order to identify and quantify sequences at a particular taxonomic level, the sequences were first grouped into “Operational Taxonomic Units” (OTUs) by clustering sequences into groups at 97% sequence identity. To account for any errors that may cause over-estimation of OTUs, firstly, an error correction step was included and involved clustering the sequences at 99% identity, resulting in 8,383,911 OTUs. Secondly, reference-based chimera detection and *de-novo* chimera detection was carried out. The number of clusters with a taxon assignment was 198,797; the majority of OTUs were assigned to bacterial taxa (196,735) with a small number of archaeal taxa (1,863) and no eukaryotic taxa due to the bias of the primers used to target the V4 region of the 16S rRNA gene (D'Amore et al., 2016). The number of OTUs for each sample (excluding samples H18-3b and L18-6c) ranged from 74,063 to 549,668, of which between 94.21 and 97.86% were aligned to a taxa (Table S2). The community composition for each sample was analyzed for each taxonomic rank (kingdom to species) (data not shown). Stacked bar plots were generated using data from QIIME showing the relative species level abundance across all samples. This was further divided into two artificial groupings, the abundant “major” species (comprising >1% of the total bacterial community within a sample) and the “minor” species (comprising < 1% of the total bacterial community within a sample).

### Canonical correspondence analysis (CCA)

Canonical Correspondence Analysis was carried out in R (v. 3.2.3, R Development Core Team, 2016) using the “vegan” package (v. 3.2.2). The environmental variables “mean temperature,” “fluctuation,” and “CO_2_” were used to determine any trends of microbial community assemblage (OTUs), with the nutrient concentrations (NH_4_, NO_x_, and PO_4_) as additional explanatory variables. A separate CCA was carried out with the same explanatory variables on larger groupings of OTUs (to either Class or Phylum) to compare trends at lower species resolution.

### Richness and diversity analysis

To negate the effect of sample size on estimating observed richness, OTU tables were repeatedly sub-sampled (rarefied) using QIIME and three measures of diversity were estimated: Chao1; the observed number of species; and the phylogenetic diversity (PD). These estimates were plotted as rarefaction curves using QIIME scripts (Figure S3). The rarefaction curves for all samples did not approach an asymptote, suggesting additional diversity could be uncovered with further sequencing. The observed number of species was defined as the number of distinct OTUs within a sample. The average observed species richness for each sample varied from 5,277 (L18-6a) to 6,181 (L18-1c) (Table S4). Chao1 (a non-parametric diversity estimator that predicts the degree to which the number of observed OTUs represented the predicted number of OTUs) was calculated to estimate richness at the species level. The average Chao1 species estimate for each sample varied from 13,425 (L12-3b) to 21,245 (L12-1b) (Table S4) (Chao, 1984; Chao and Lee, 1992; Colwell and Coddington, 1994). When comparing the difference between observed and predicted (Chao1) OTU diversity, sample L12-3b had the smallest difference (8,113 OTUs) representing a total of 39.6% of the predicted OTUs and L12-1b had the highest difference (15,160 OTUs) representing 28.6% of the predicted OTUs. The phylogenetic diversity (PD) represents the minimum total branch length that covers all taxa within a sample on a phylogenetic tree, therefore a smaller value indicates a reduced expected taxonomic diversity, whilst a larger value indicates a higher expected diversity (Faith, 1992). The PD values for this dataset ranged from 323 (L18-6a) to 434 (H18-6c).

To compare the similarity of samples based on the bacterial sequences, the distance between each sample was calculated using a UniFrac metric. The distance was defined as the sum of the unshared branch lengths between two samples divided by the total branch length (shared and unshared) of two samples (Lozupone and Knight, 2005). The fraction of the branch length unique to each sample was then calculated (i.e., the lower this value, the more similar the two samples are) using weighted UniFrac distances which takes into account OTU abundance and branch weights accordingly (as opposed to an unweighted Unifrac distance which would consider only OTU presence/absence) (Lozupone and Knight, 2005, 2007).

## Results

### Metagenomic analysis

**Figure 1:**
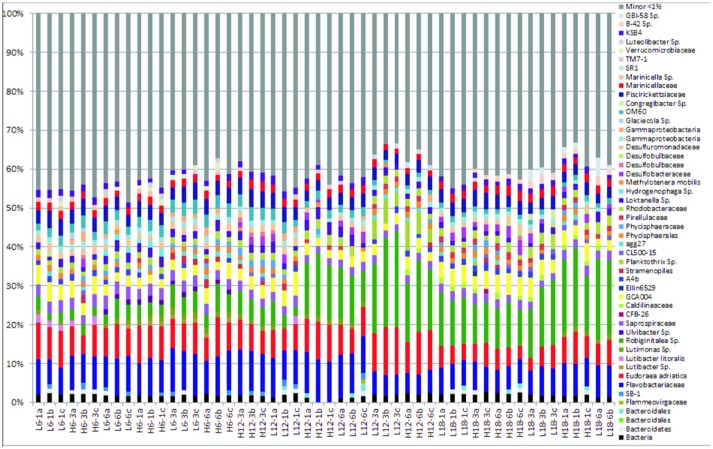
Relative abundance of bacterial community compositions for 52 sediment samples at species level, including taxonomic identification for only sequences that comprised >1% of the total bacterial community for each sample. “Minor” species are all species that comprise < 1% of the total bacterial community for each sample, which have been artificially clustered together into a “minor” species group, depicted by the gray coloring.

The “minor” species were observed to comprise between 35–45% of the total abundance across all samples and environmental regimes (Figure 1, Figure S2). In contrast, the “major” species dominated relative abundance, ranging from 55 to 75% of relative abundance within each sample (Figure 1). A summary table of all major species (OTUs) listed by taxon and treatment can be found in the Supplementary Information (Table S3).

### Microbial response to environmental variables

**Figure 2:**
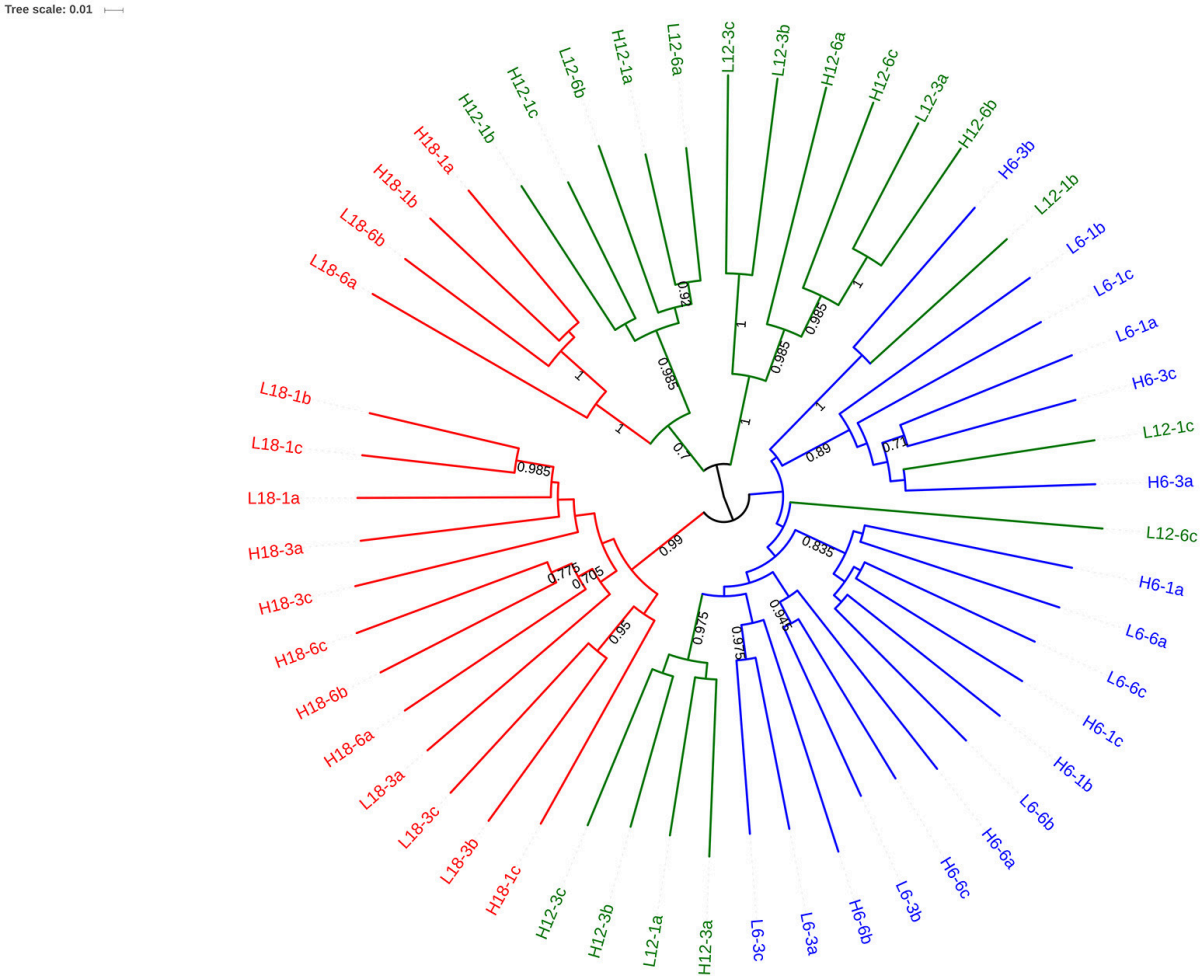
UPGMA tree with Jacknife support using weighted Unifac distance. Nodes with >0.8 Jacknife support are labeled. Branches are color coded to reflect the mean temperature of the experimental regime: 6°C (blue), 12°C (red) and 18°C (green). The labels of each branch correspond to environmental conditions.

**Figure 3:**
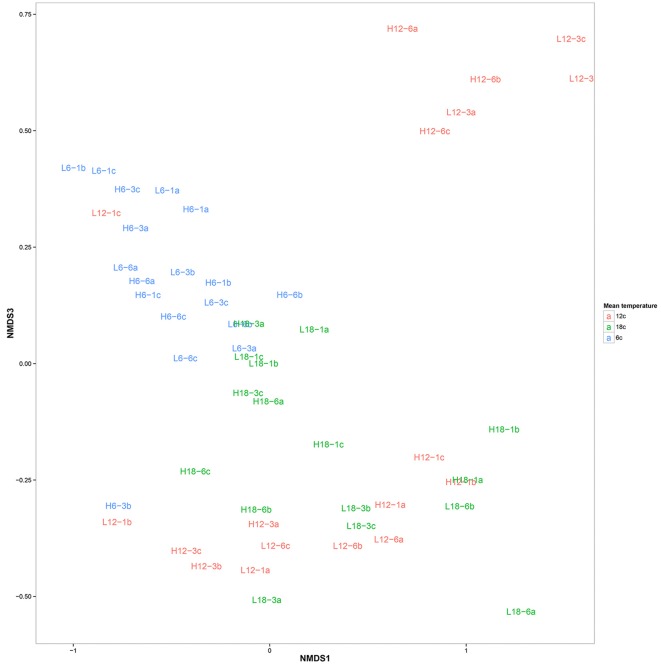
Non-metric multidimensional scaling plot of bacterial community composition color coded according to mean temperature: 6°C (blue), 12°C (red), and 18°C (green). The labels correspond to environmental conditions.

OTU clustering was visualized using an Unweighted Pair Group Method with Arithmetic Mean (UPGMA) tree (Figure 2) with Jacknife support (Sokal and Michener, 1958). The dissimilarity matrix generated for the Unifrac metric (data not shown) was also utilized for non-metric multidimensional scaling (NMDS) analysis to visualize (in plot form) the sequence data with respect to the environmental variables including; mean temperature (Figure 3), CO_2_ treatment and temperature fluctuation (Figure S4). From both the UPGMA tree grouping (Figure 2) and NMDS plots, a strong mean temperature effect on species-level bacterial community composition was observed, as reflected by sample grouping in relation to mean temperature (6, 12, and 18°C). Nutrient concentration for PO_4_ and NO_x_ was consistently low, but NH_4_ varied with mean temperature, not CO_2_ or temperature fluctuation (Figure 5). The nutrient concentration data was included for analysis with microbial community assemblages. PO_4_ concentration decreased over time, which appears to correspond with a reduction in the abundance of *Gammaproteobacteria* (see section Species-specific microbial responses).

**Figure 4:**
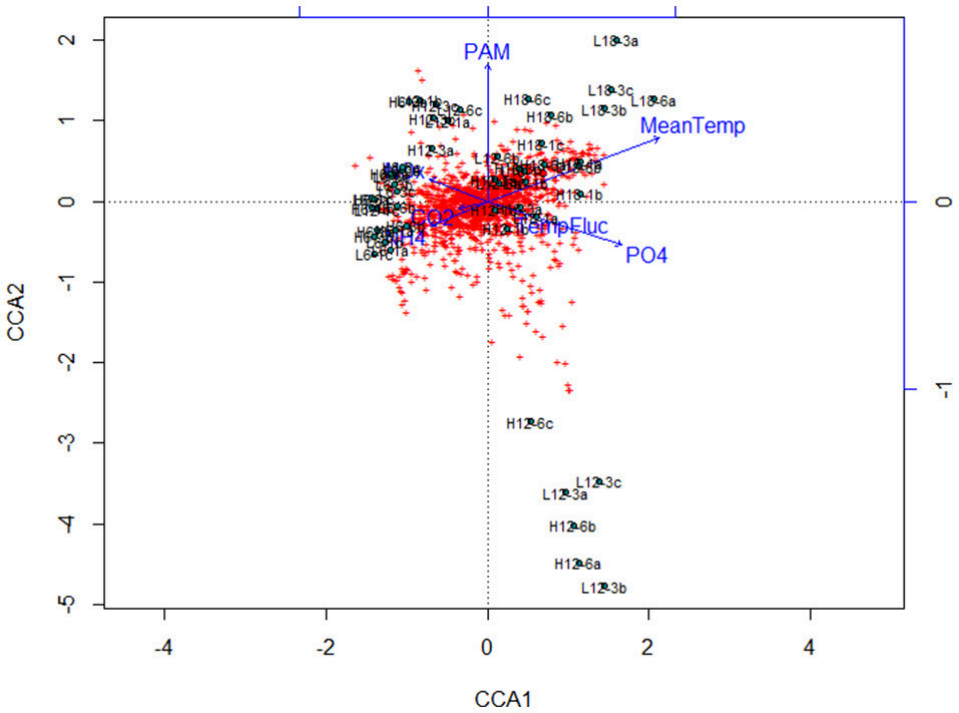
Canonical Correspondence Analysis (CCA) plot for major bacterial species (comprising >1% of the total bacterial community composition of each sample) at species level (OTU) resolution. The blue lines and labels correspond to the environmental conditions and nutrient concentrations, and the black labels represent the individual treatments.

**Figure 5:**
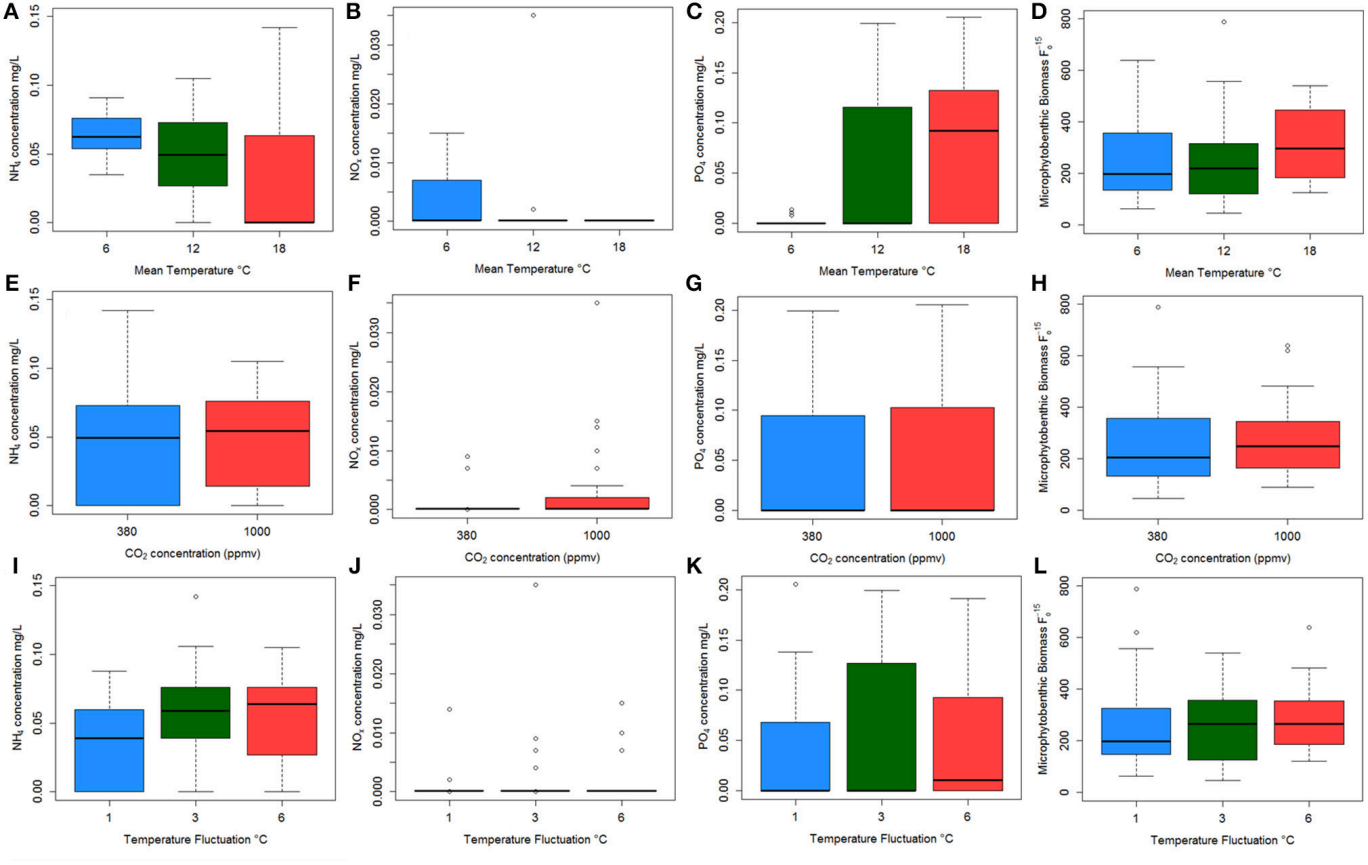
Boxplots showing the raw data for each nutrient concentration **(A–I)** and microphytobenthos (MPB) biomass **(J–L)** against each environmental: NH_4_ concentration **(A,E,I)**; NO_x_ concentration **(B,F,J)**; PO_4_ concentration **(C,G,K)** and MPB biomass **(D,H,L)** against mean temperature (top plots); CO_2_ regime (middle plots); and temperature fluctuation (bottom plots). Colors indicate mean temperature treatments of 6°C (blue), 12°C (green), and 18°C (red) in the top three graphs; represent CO_2_ levels of 380 ppmv (blue) and 1,000 ppmv (red) in the middle plots; and temperature fluctuation of 1°C (blue), 3°C (green), and 6°C (red).

**Figure 6:**
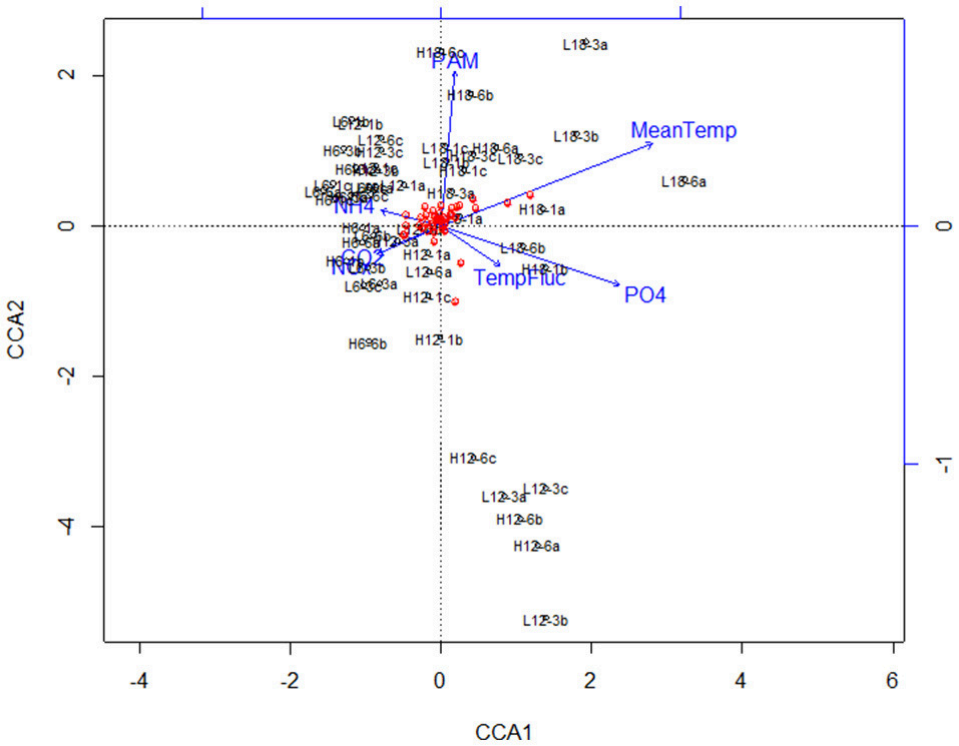
Canonical Correspondence Analysis (CCA) plot for major bacterial species (comprising >1% of the total bacterial community composition of each sample) at class/order resolution. The blue lines and labels correspond to the environmental conditions and nutrient concentrations, and the black labels represent the individual treatments.

Canonical correspondence analysis (CCA) examined the effect of environmental variables on the bacterial community composition. Using the species (OTU) level bacterial composition (note: the few archaea sequences detected were excluded, the primers chosen target the V4 region of the 16S rRNA gene as bacteria were the focus of this study), mean temperature (*F* = 18.7059, *p* = 0.005), MPB community (*F* = 4.4852, *p* = 0.01) and PO_4_ concentration (*F* = 4.0939, *p* = 0.020) was found to significantly influence the species level bacterial community composition (Figure 4). This CCA explained 42% of the variance, and the effects of CO_2_ and fluctuating regime were not significant. The variations in ammonium (NH_4_) and nitrate-nitrite (NO_x_) concentrations were also insignificant (NH_4_: *F* = 1.8149, *p* = 0.01; NO_x_: *F* = 1.6684, *p* = 0.1) in the CCA. The raw data for each nutrient concentration is presented in boxplots according to treatment, and did not change with mean temperature, CO_2_ or fluctuating regime (Figure 5). The CCA was rerun using OTUs which were grouped by either Phyla or Class, based on the lowest resolution by clustering. Although similar trends were observed, the variance explained was lower (39%). Mean temperature was the most significant variable (*F* = 12.7389, *p* = 0.005), followed by PO_4_ concentration (*F* = 4.2576, *p* = 0.01) and MPB biomass (*F* = 4.3762, *p* = 0.03). CO_2_, fluctuating regime and NH_4_ and NO_x_ concentration were again found to have no significant effect (Figure 6).

### Species-specific microbial responses

To assess the bacterial species responsible for driving observed changes influenced by mean temperature (Figures 2–**4**), the species level community composition under each of the three experimental temperatures were compared (Table 2). Microbial communities were dominated by Bacteroidetes (predominantly the family *Flavobacteriae*), with over 50% of total relative abundance attributed to this phylum across all mean temperature treatments, peaking at 59% at 12°C (Figure 1, Table 2). Although the overall Bacteroidetes relative abundance was fairly constant, the community structure within *Flavobacteria* changed with mean temperature. For example, at genus level *Robiginitalea* increased in abundance as mean temperature increased, from 7% at 6°C to 20% at 12°C and 23% at 18°C. In contrast, *Lutibacter* species (*L. litoralis* and *Lutibacter* spp.) were both absent at 18°C mean temperature, and *Lutibacter* spp. were only found under the 6°C mean temperature treatments (Table 2). *Ulvibacter* sp. was only present at 6°C, and *Eudorea adriatica* declined from 14% relative abundance at 6°C and 12°C to 9% at 18°C.

**Table 2:** Changes in abundance at class, order and family level under the key phyla in the “major species” group, classified to the lowest taxonomic level, under each mean temperature regime.

**Phylum**	**Class**	**Order**	**Family**	**Genus**	**Species**	**Average strains (% relative abundance)**		
						**6**°**C**	**12**°**C**	**18**°**C**
*Planctomycetes*	–	–	–	–	–	*4,965 (4.4%)*	*9,456 (4.7%)*	*7,432 (6.5%)*
	*Phycisphaerae*	*Phycisphaerales*	–	–	–	2,487 (2.1%)	2,220 (1.7%)	2,683 (2%)
	*Phycisphaerae*	*Phycisphaerales*	*Phycisphaeraceae*	–	–	1,243 (1.1%)	1,466 (1%)	1,044 (0.8%)
	*Planctomycetia*	*Pirellulales*	*Pirellulaceae*	–	–	237 (0.2%)	1,111 (0.8%)	2,918 (2%)
*Bacteroidetes*	–	–	–	–	–	*5,7749 (51%)*	*7,4825 (59%)*	*75,165 (53%)*
	*Flavobacteria*	*Flavobacteriales*	*Flavobacteriaceae*	*-*	–	46,500 (41%)	64,482 (50%)	66,365 (45%)
	–	–	–	*Robiginitalea*	–	7,334 (7%)	25,602 (20%)	35,071 (23%)
	–	–	–	*Lutimonas*	–	2,166 (1.9%)	1,691 (1.3%)	1,012 (0.7%)
	–	–	–	*Lutibacter (excl litoralis)*	–	1,002 (0.9%)	0 (0%)	0 (0%)
	–	–	–	*Lutibacter*	*litoralis*	1,053 (0.9%)	230 (0.2%)	0 (0%)
	–	–	–	*Eudoraea*	*adriatica*	1,5723 (14%)	18,087 (14%)	12,902 (9%)
	–	–	–	*Ulvibacter*	–	1,429 (1.3%)	0 (0%)	0 (0%)
	*Sphingobacteria*	*Sphingobacteriales*	*Saprospiraceae*	–	–	5856 (5.2%)	4697 (3.6%)	5516 (3.9%)
*Cyanobacteria*	–	–	–	–	–	*1290 (1.1%)*	*802 (3.3%)*	*246 (0.3%)*
	*Oscillatoriophycidaea*	*Oscillatoriales*	*Phormidiaceae*	*Planktothrix*	–	0 (0%)	3 (2.7%)	0.1 (0.1%)
*Proteobacteria*	–	–	–	–	–	*34,331 (30.5%)*	*33,108 (25.3%)*	*33,651 (23.4%)*
	*Alphaproteobacteria*	–	–	–	–	6,874 (6.2%)	8,672 (6.6%)	8,700 (5.8%)
	*Alphaproteobacteria*	*Rhodobacterales*	*Rhodobacteraceae*	–	–	3,056 (2.7%)	4,113 (3.1%)	5,272 (3.5%)
	–	–	–	*Loktanella*	–	3,818 (3.5%)	4,559 (3.5%)	3,428 (2.3%)
	*Betaproteobacteria*	–	–	–	–	*1,084 (1%)*	*456 (0.3%)*	*0 (0%)*
	*Betaproteobacteria*	*Burkholderiales*	*Comamonadaceaea*	*Hdrogenophaga*	–	970 (0.9%)	0 (0%)	0 (0%)
	–	*Methylophilales*	*Mthylophilaceae*	*Methylotenera*	*mobilis*	115 (0.1%)	456 (0.3%)	0 (0%)
	*Deltaproteobacteria*	–	–	–	–	*3,829 (3.3%)*	*4,862 (3.6%)*	*5,751 (4.1%)*
	–	*Desulfobacterales*	*Desulfobacteraceae*	*Desulfobacteraceae*	–	290 (0.2%)	2,262 (1.7%)	3,350 (2.4%)
	–	*Desulfobacterales*	*Desulfobulbaceae*	*Desulfobulbaceae*	–	136 (0.1%)	603 (0.4%)	163 (0.1%)
	–	*Desulfobacterales*	*Desulfobulbaceae*	*Desulfobulbaceae*	–	114 (0.1%)	0 (0%)	670 (0.4%)
	–	*Desulfouoromonadales*	*Desulfuromonadaceae*	*Desulfuromonadaceae*	–	3,288 (2.9%)	1,997 (1.5%)	1,570 (1.2%)
	*Gammaproteobacteria*	–	–	–	–	*22543 (20%)*	*19119 (15%)*	*19200 (14%)*
	–	*Other*	–	–	–	3,040 (3.7%)	4,373 (2.4%)	3,029 (2.1%)
	–	*Alteromonadales*	*OM60*	–	–	5,562 (5.01%)	4,373 (3.3%)	2,800 (2%)
	–	*Alteromonadales*	*OM60*	*Congregibacter*	–	0 (0%)	0 (0%)	539 (0.4%)
	–	*Thiotrichales*	*Piscirickettsiaceae*	–	–	7,585 (6.7%)	7,408 (5.6%)	7,691 (5.5%)
	–	*Marinicellales*	*Marinicellaceae*	–	–	3,960 (3.5%)	4,166 (3.2%)	5,141 (3.6%)
	–	*Marinicellales*	*Marinicellaceae*	*Marinicella*	–	1,059 (1%)	132 (0.1%)	0 (0%)
*Deinococcus–Thermus*	*Deinococci*	*Deinococcales*	*Trueperaceae*	–	–	**0 (0%)**	**0 (0%)**	**5,895 (3.2%)**
	*Deinococci*	*Deinococcales*	*Trueperaceae*	*B−42*	–	0 (0%)	0 (0%)	2,990 (1.9%)
	*Deinococci*	*Deinococcales*	*Trueperaceae*	*GBI−58*	–	0 (0%)	0 (0%)	2,905 (1.3%)
*Verrucomicrobia*	–	–	–	–	*-*	**1,995 (1.8%)**	**371 (0.3%)**	**0 (0%)**
	*Verrucomicrobiae*	*Verrucomicrobiales*	*Verrucomicrobiaceae*	–	–	1,335 (1.2%)	0 (0%)	0 (0%)
	*Verrucomicrobiae*	*Verrucomicrobiales*	*Verrucomicrobiaceae*	*Luteolibacter*	–	660 (0.6%)	371 (0.3%)	0 (0%)

*The values represent the total number of OTUs across all replicates (n = 3), with % relative abundance in brackets. Values in italics and underlined represent the total values for each phylum*.

Proteobacteria was the second most abundant phylum after Bacteroidetes, making up 30% of relative abundance at 6°C, but dropping to 25% at 12°C and 23% at 18°C. The *Gammaproteobacteria* class underpinned the decreasing trend found in the Proteobacteria phylum, decreasing in abundance from 20% at the lowest mean temperature 6°C to 15 and 14% at 12° and 18°C respectively (Table 2). *Betaproteobacteria* were present at the lower temperature treatments, but were not found in the highest mean temperature treatment (18°C). In contrast, *Alphaproteobacteria* remained relatively constant (~6%) across all treatments, and *Deltaproteobacteria* increased in abundance with increasing mean temperature, so although the overall phylum abundance decreased, this observation masked changes in the lower taxonomic levels.

The phylum Planktomycete*s* was present in all samples, with an average constant abundance of ~5% which increased slightly as mean temperature increased (Figure 1, Table 2). However, as observed with *Flavobacteria*, the overall abundance masks individual species level dynamics, with *Phycisphaerae* (~2%) and *Phycisphaerales* (~1%) dominating the lower temperature regimes within this phylum. However, as mean temperature increases, the relative abundance of *Pirellulaceae* increases, rising from 0.2% at 6°C to 2% at 18°C. In general, Cyanobacteria abundance was low in all samples; with Cyanobacteria sp. found at all mean temperatures. As observed for other phyla, this general trend was underpinned by specific species dynamics. At a mean temperature of 12°C (specifically L12-3 and H12-6, Figure 1), there was a large relative abundance (up to 12%) of the cyanobacterium *Planktothrix*, which was only found in one other treatment (H18-3a). This was also reflected where samples with *Planktothrix* at the mean temperature of 12°C were clustered together (Figure 3). *Verrucomicrobia* showed distinct temperature response dynamics, decreasing in abundance as mean temperature increases, with no *Verrucomicrobia* present at 18°C. In contrast, thermophilic bacteria from the phylum Deinococcus-Thermus were found only in the highest mean temperature regime (Table 2).

## Discussion

There is clear evidence of environmental change affecting species distributions and abundances, and this changing biodiversity has been well studied in benthic systems, through a variety of manipulative experiments and observational studies (Ieno et al., 2006; Prosser et al., 2007; Bulling et al., 2010; Hicks et al., 2011; Gilbertson et al., 2012; Godbold and Solan, 2013). However, most of these studies focus on macrofaunal diversity, although it is the microbial assemblages in these habitats that drive biogeochemical cycling (Middelburg, 2011; Mayor et al., 2012). Studies that examine shifts in microbial communities in relation to environmental changes have tended to focus on only one environmental variable, such as CO_2_ gradients (Kerfahi et al., 2014; Tait et al., 2015) and the impact on relative class or order level abundance (Tait et al., 2013, 2015); or targeting specific genes, for their biogeochemical properties (Kitidis et al., 2017). This study generated over 11 million sequences, with taxonomic identification achievable at species level (97% sequence identity). The number of OTUs found through NGS was much higher than numbers found using T-RFLP, ARISA, or DGGE (Massé et al., 2016), and provided greater resolution on species level changes that may be masked in studies that sequence to class/order, or only provide information on overall bacterial biomass (Mayor et al., 2013; Main et al., 2015). The comparison of different resolution (class/order analysis compared to species level analysis) showed the same trends, but a lower species resolution may not only mask species level changes, but also miss interactions between environmental variables. In future, it would be interesting to compare the results observed with direct sequencing of rRNA as it has been shown to eliminate uncertainties associated with primer matching on the rDNA and therefore producing a more robust assessment of bacterial populations (Rosselli et al., 2016).

Benthic microbes play a vital role in sediment biogeochemistry (Bertics and Ziebis, 2009), and their contribution to ecosystem function is determined by community assemblage (Petchey and Gaston, 2006; Beveridge et al., 2010). This study supports previous research on coastal sediments, which has shown that Proteobacteria (alpha, beta, delta, and gamma), Bacteroidetes, and Planctomycetes dominate relative abundance (Musat et al., 2006; Laverock et al., 2010; Gobet et al., 2012; Tait et al., 2015). Overall relative abundance did not change at class or order level in response to changes in CO_2_, as seen in previous manipulative research (Tait et al., 2013, 2015), although microbial community changes have been found along a natural CO_2_ gradient in the Mediterranean (Kerfahi et al., 2014). This study found that changes in mean temperature, not CO_2_, have a significant effect on shifts in microbial community assemblage, and the contribution of certain taxa to specific ecosystem services (such as nutrient cycling) may be altered with environmental change, particularly with warming temperature (Bertics and Ziebis, 2009). Results indicate that this varies between orders and classes, with some remaining constant in relative abundance (e.g., *Flavobacteria*), supporting previous work (Musat et al., 2006; Laverock et al., 2010; Gobet et al., 2012), and others such as the Proteobacteria changing in abundance with increased mean temperature. However, this study illustrates the apparent constant abundance may conceal changes in community structure at genus or species taxonomic levels as a result of the level of detail provided by next generation sequencing.

Microbial communities play a vital role in benthic carbon cycling and are often the primary degraders of organic matter when it reaches the sediment surface. Bacteroidetes are important for initial organic matter degradation, often breaking down complex polymeric substances (Teeling et al., 2012; McKew et al., 2013; Taylor et al., 2013; Decleyre et al., 2015). The microphytobenthic (MPB)-rich sediment used in this study are typical of tidal mudflats, and the extracellular polymeric substances excreted by MPB provide a labile carbon source for heterotrophic microorganisms (McKew et al., 2013; Taylor et al., 2013; Decleyre et al., 2015; Bohorquez et al., 2017). Bacteroidetes are the dominant phylum here, in particular *Flavobacteria* (which make up 80% of the Bacteroidetes abundance), and together with Plactomycetes, they play a vital role in benthic carbon cycling as the initial organic matter degraders (McKew et al., 2013; Taylor et al., 2013; Bohorquez et al., 2017). Despite the changing environmental conditions, their relatively constant abundance suggests the initial degradation of carbon remains unaffected by temperature changes, perhaps unsurprising as many tidal benthic species are facultative anaerobes (McKew et al., 2013). Although the relative abundance of the *Flavobacteria* remains constant, there are changes in the community structure with increasing temperature, such as an increase in *Robiginitalea* as mean temperature increases (which corresponds to an increase in PO_4_), and a corresponding decrease in *Eudoraea adriatica* and *Lutibacter* species (*L*. *litoralis* is only found at 6°C mean temperature treatment). Species within the *Robiginitalea* genus are known to have a thermal preference above 10–15°C (Cho and Giovannoni, 2004; Manh et al., 2008), which may explain why they increase from 7% at 6°C mean temperature to 23% at 18°C, thus maintaining the overall constant relative abundance of the *Flavobacteriaceeae* family as the *Lutibacter* and *Eudoraea* species decline with rising mean temperature. This maintains the functionality of this group as primary carbon degraders, although the species within the family that carry out this process have shifted with increasing temperature, suggesting some redundancy with in the *Flavobacteria*.

In the dominant phylum Bacteroidetes, a decrease in the *Saprospiraceae* family was observed with increasing mean temperature, which has implications for the carbon cycle, as they are dominant in coastal zones and play an important role in remineralisation of organic matter (Raulf et al., 2015). Previous studies have suggested that *Saprospiraceae* strains are sensitive to environmental changes, although in this study a temperature effect was demonstrated, not a shift due to elevated CO_2_ (Raulf et al., 2015).

It is also possible that these species level changes may cause a shift in the function or capability within a bacterial class or order, although the overall abundance of a class may remain constant, as found for the *Flavobacteria* (Table 2). The change in nutrient concentration for (decreasing) ammonia (NH_4_) and (increasing) phosphate (PO_4_) with increasing mean temperature support this concept. Here we demonstrate an increase in sulfate reducing bacteria (*Deltaproteobacteria*) as mean temperature increases, and the presence of thermophilic bacteria (*Deinococcus-Thermus*) at the highest mean temperature treatment (18°C). Sulfate reducing bacteria (SRB) are often found in cohesive sediments (Ravenschlag et al., 2000), such as the intertidal muddy sediment used in this study, due to the steep redox gradients determined by the permeability and oxygen penetration depth (Probandt et al., 2017). Sulfate reducers are associated with anoxic sediment (Orcutt et al., 2011), and the increase in SRB abundance with increasing temperature may also be indicative of lower oxygen concentrations with the warming regimes, driving the redox layer toward the sediment surface and promoting formation of anoxic “hotspots” within the sediment, stimulating sulfate reduction (Mahmoudi et al., 2015). There were clear visual differences in the highest mean temperature treatments, with the sediment profile in the mesocosms turning from an oxic brown color to black, suggesting the redox layer has shifted closer to the sediment surface, supporting sulfate reducing conditions. As strict anaerobes, *Desulfobacteraceae* remineralise organic matter in the absence of oxygen (Probandt et al., 2017), and are often found in fine impermeable sediments which promote the development of anoxic niches within the surface sediments, enhanced by the higher mean temperature in this study. A corresponding increase in the abundance of extremophilic species (Deinococcus-Thermus phylum), typically found in harsh environments such as deserts and hot springs (Albuquerque et al., 2005; Pikuta et al., 2007), was also measured in the highest mean temperature regime. This demonstrates the shifting regime in the benthic microbial community at a genus and species level, and the consequent shift from aerobic processes to favoring anaerobic processes in the sediment surface.

Previous work has demonstrated that stable environmental conditions promotes constant and specific microbial communities (Bertics and Ziebis, 2009), but it is unclear how quickly these communities may respond to change. The interpretation of change in microbial communities is dependent on the depth of diversity measured (e.g., down to genus or species level or identifying classes or orders). However, while species turnover may be obvious when using the higher taxonomic resolution, lack of turnover does not necessarily result in static functionality. Freshwater microbial communities are often characterized by their metabolic plasticity in response to environmental change, which contributes to their functional redundancy and links their assemblage composition with ecosystem function (Comte et al., 2013). In the present study we demonstrate a clear response in the marine benthic microbial community to different mean temperature treatments that would have been overlooked using poorer taxonomic resolution. This changing community reflected a change in nutrient concentrations as mean temperature increased, thus suggesting there is no functional redundancy among the different species which provides resilience to environmental change (Muntadas et al., 2016). However, in terms of carbon cycling, there is a shift in the community assemblage within the *Flavobacteria*, the relative abundance remains constant, suggesting some functional redundancy with organic matter degradation. Much of the nitrogen cycle is driven by archaea (Raulf et al., 2015), such as ammonia-oxidizing archaea (AOA), which were not measured in this study due to the bacterial specific primers used. However, ammonia oxidizing bacteria (AOB), predominantly affiliated with Betaproteobacteria (β-AOB) (Bernhard et al., 2005), play a significant role in nitrogen cycling (Risgaard-Petersen et al., 2004), and can outnumber AOA in coastal sediments (Santoro et al., 2008). In this study, increasing mean temperature led to a decrease in Betaproteobacteria abundance, with no Betaproteobacteria present at the highest mean temperature. Although ammonia oxidisers were identified (both *Nitrosomonas* and *Nitrospora*) within the Betaproteobacteria, their abundance was less than 1% across all treatments. The corresponding decrease in NH_4_ concentration in the overlying water suggests there may be changes in the nitrogen cycling, possibly influenced by the absence of Betaproteobacteria, and NO_x_ levels remain low across all treatments (Table 2). The phosphate increase could be linked to the corresponding decrease in abundance of Gammaproteobacteria, which are instrumental in phosphorous cycling (Sebastian and Gasol, 2013) and are usually limited by phosphate availability, and there is a corresponding increase in the abundance of *Robiginitalea*. The decrease in Gammaproteobacteria means the uptake of phosphate from the overlying water column is reduced, leading to the rising concentrations with rising temperature, directly impacting the phosphorous cycling in this benthic system. In addition, changes in the redox layer in the surface sediment will release iron-bound phosphorous under anoxic conditions (Sinkko et al., 2011), enhancing overall phosphorous flux from the sediment into the water. Ammonium and phosphate are typically the preferred nutrients for microbial communities, and the consistently low nitrate (and nitrite) concentrations in this study are typical of coastal oligotrophic systems (Chen et al., 2017). The change in NH_4_ concentration may be a result of a combination of low abundance of ammonia oxidizer bacteria, reduced microphytobenthos activity or a higher rate of microbial community mineralisation with increasing mean temperature.

In conclusion, changes in microbial assemblage were only found between the mean temperature treatment, and not in response to changes in diurnal temperature variability or elevated CO_2_. This supports recent research that has shown seasonal changes mask any response to elevated CO_2_ within the environment (Tait et al., 2013, 2015; Currie et al., 2017; Hicks et al., 2017b). However, some of the changes at species level, such as increasing abundance of sulfate reducing bacteria (*Desulfobacteraceae*) and corresponding decrease of *Desulfuromonadaceae* with increasing mean temperature, suggest that changes to the sulfur cycle may not be noticed at the ecosystem service level, despite a change in species assemblage. Targeted future work should address how changes in some species (e.g., increase of thermophilic species in the *Deinococcus-Thermus* phylum) may be reflected in a broad range of biogeochemical processes, such as integrating measurements relating to sulfur, nitrogen and carbon cycles. Sediment profiles of oxygen and H_2_S would provide insight into potential shifting from oxic to anoxic (sulfate reduction) conditions, and this linked to corresponding microbial communities would provide direct biogeochemical information on coastal sediment dynamics. This study has focused on intertidal cohesive sediments, but the microbial response will vary with sediment type, driven by changes in oxygen penetration depth (Hicks et al., 2017a). The depth of taxonomic resolution provided by NGS provides additional information at a genus or species level, allowing identification of species regime shifts that may directly impact biogeochemistry, which may be missed using a lower taxonomic resolution technique. High taxonomic resolution is useful for identifying species shifts and measuring potential functional redundancy for key biogeochemical processes, such as carbon mineralization and nutrient cycling. Since benthic systems provide a variety of ecosystem services (Duffy and Stachowicz, 2006; Frid and Caswell, 2016) which are often driven by microbial activity, these results suggest some vulnerability (nutrients), and highlights potential functional redundancy (carbon), in benthic microbial communities as a response to climate changes. Importantly, elevated CO_2_ does not appear to have any effect on microbial assemblage, based on the results discussed here, although changing mean temperature (and not variability) appears to drive community assemblage change. Whilst there are limitations in realistically interpreting results from artificial mesocosm systems, and caution must be taken in interpreting responses, these experiments are valuable in providing insights on how complex ecosystems may respond to warming or elevated CO_2_ (Benton et al., 2007; Cartaxana et al., 2015). This has implications for environmental change research, particularly in coastal habitats where much of the ecosystem services are generated through microbial interactions that occur in the benthos. Changes to nutrient cycling (such as the availability of nitrogen or phosphate) could promote eutrophication or decrease phytoplankton primary production (Vitousek et al., 1997), directly impacting food webs and indirectly affecting benthic carbon mineralization and sequestration. Integrating next generation sequencing with robust biogeochemical parameters is key in understanding the potential consequences of environmental change in coastal habitats.

## Author contributions

NH and DP conceived and designed the experiments. NH ran the experiments and collected the samples. NH and KD performed DNA extraction using a protocol developed. KD, XL, RG, JK, AL, and LL ran the bioinformatics, including sampling, quality control, data processing and sequence assignment. XL performed additional analysis on genomic results. NH, KD, and DP wrote the manuscript, with input from all co-authors.

### Conflict of interest statement

The authors declare that the research was conducted in the absence of any commercial or financial relationships that could be construed as a potential conflict of interest.
