# Supplementary material for: Temperature Driven Changes in Benthic Bacterial Diversity Influences Biogeochemical Cycling in Coastal Sediments
Source: Front Microbiol. 2018 Aug 22;9:1730. doi: 10.3389/fmicb.2018.01730 (PMC6115492; doi:10.3389/fmicb.2018.01730)
Supplement: Supplementary file 1 [file Data_Sheet_1.pdf]

Supplementary Material

Temperature driven changes in benthic microbial diversity influences biogeochemical cycling under future climate scenarios

Natalie Hicks[1*], Xuan Liu[2], Richard Gregory[2], John Kenny[2], Anita Lucaci[2], Luca Lenzi[2], David M. Paterson[3], Katherine R. Duncan[4*]

[1] The Scottish Association for Marine Science, Scottish Marine Institute, Oban. Argyll, PA37 1QA, UK

[2] Centre for Genomic Research, Institute of Integrative Biology, Biosciences Building, University of Liverpool, Liverpool, L69 7ZB, UK

[3] Sediment Ecology Research Group, Scottish Oceans Institute, School of Biology, University of St Andrews, Fife, KY16 8LB, UK

[4] University of Strathclyde, Strathclyde Institute of Pharmacy and Biomedical Sciences, 161 Cathedral Street, Glasgow, G4 0RE, UK

Correspondence:
Natalie Hicks, natalie.hicks@sams.ac.uk;
Katherine Duncan, katherine.duncan@strath.ac.uk

Figure S1: **(a)** Experimental design showing 18 unique treatments (i) two CO_2 treatments; (ii) three mean temperatures; and (iii) three fluctuation regimes which were repeated in triplicate to yield 54 samples and **(b)** mean temperature and temporal variability of environmental regimes, each 24 hour period included one complete fluctuation cycle above and below the mean.

(a)

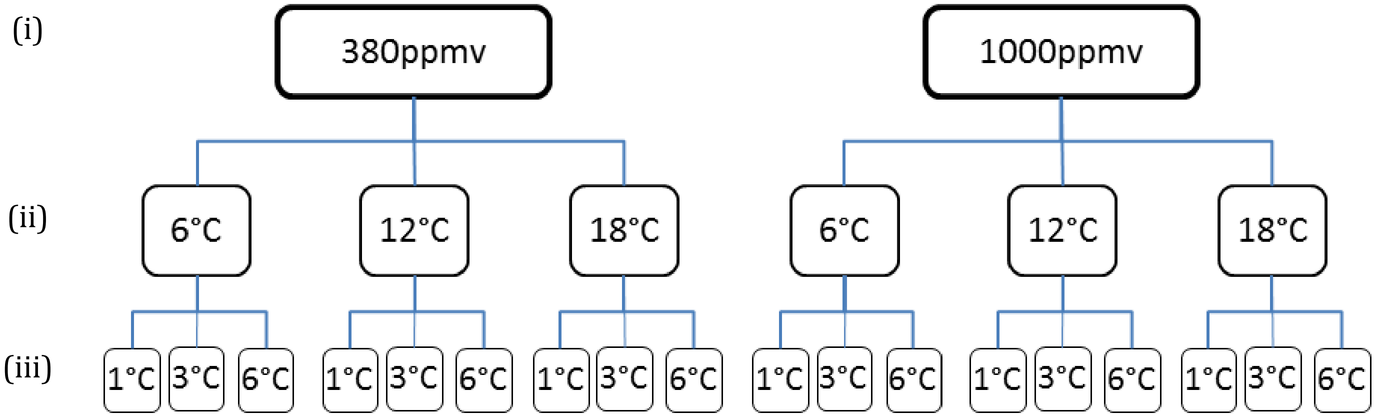

(b)

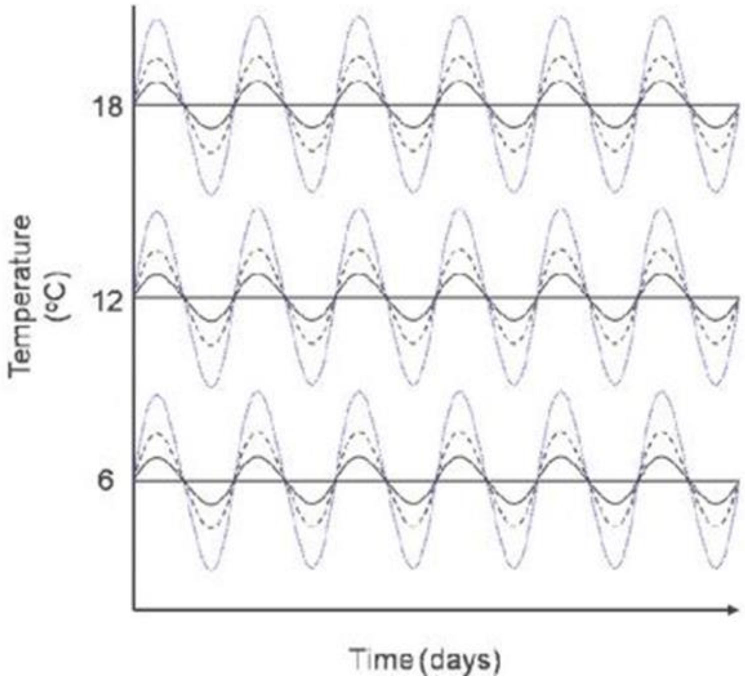

Table S1: Summary of sequence data before and after adapter and quality trimming, showing forward (R1) and reverse (R2) read pairs and singlet (RO) reads for all 54 sediment samples.

Treatment	Nextera Dual Index	Raw Reads	Trimmed Reads[1] (%)[2]	R1/R2 Read Pairs [1]	R0 Reads[1] (%)[3]
L6-1a	GTAGAGGA-TAGATCGC	470,438	469,841 (99.87)	234,628	585 (0.12)
L6-1b	GTAGAGGA-CTCTCTAT	416,692	416,254 (99.89)	207,908	438 (0.11)
L6-1c	GTAGAGGA-TATCCTCT	449,666	449,201 (99.90)	224,368	465 (0.10)
H6-3a	GTAGAGGA-AGAGTAGA	473,564	472,651 (99.81)	236,053	545 (0.12)
H6-3b	GTAGAGGA-GTAAGGAG	497,076	496,230 (99.83)	247,830	570 (0.11)
H6-3c	GTAGAGGA-ACTGCATA	409,498	409,117 (99.91)	204,369	379 (0.09)
L6-6a	GTAGAGGA-AAGGAGTA	354,918	354,205 (99.80)	176,884	437 (0.12)
L6-6b	GTAGAGGA-CTAAGCCT	376,416	375,939 (99.87)	187,732	475 (0.13)
L6-6c	AAGAGGCA-TAGATCGC	416,744	416,276 (99.89)	207,906	464 (0.11)
H6-1a	AAGAGGCA-CTCTCTAT	373,780	373,436 (99.91)	186,547	342 (0.09)
H6-1b	AAGAGGCA-TATCCTCT	384,914	384,526 (99.90)	192,070	386 (0.10)
H6-1c	AAGAGGCA-AGAGTAGA	349,680	348,984 (99.80)	174,291	402 (0.12)
L6-3a	AAGAGGCA-GTAAGGAG	422,000	421,264 (99.83)	210,399	466 (0.11)
L6-3b	AAGAGGCA-ACTGCATA	357,616	357,277 (99.91)	178,469	339 (0.09)
L6-3c	AAGAGGCA-AAGGAGTA	483,136	482,291 (99.83)	240,897	497 (0.10)
H6-6a	AAGAGGCA-CTAAGCCT	430,026	429,583 (99.90)	214,570	443 (0.10)
H6-6b	CGAGGCTG-TAGATCGC	346,426	346,044 (99.89)	172,834	376 (0.11)
H6-6c	CGAGGCTG-CTCTCTAT	456,078	455,587 (99.89)	227,548	491 (0.11)
H12-3a	CGAGGCTG-TATCCTCT	751,066	750,278 (99.90)	374,748	782 (0.10)
H12-3b	CGAGGCTG-AGAGTAGA	463,930	463,358 (99.88)	231,398	562 (0.12)
H12-3c	CGAGGCTG-GTAAGGAG	472,896	472,342 (99.88)	235,908	526 (0.11)
L12-1a	CGAGGCTG-ACTGCATA	402,448	401,995 (99.89)	200,773	449 (0.11)
L12-1b	CGAGGCTG-AAGGAGTA	467,026	466,435 (99.87)	232,927	581 (0.12)
L12-1c	CGAGGCTG-CTAAGCCT	405,130	404,605 (99.87)	202,044	517 (0.13)
H12-1a	GCTACGCT-TAGATCGC	421,538	421,173 (99.91)	210,408	357 (0.08)
H12-1b	GCTACGCT-CTCTCTAT	449,600	449,215 (99.91)	224,416	383 (0.09)
H12-1c	GCTACGCT-TATCCTCT	422,858	422,520 (99.92)	211,092	336 (0.08)
L12-6a	GCTACGCT-AGAGTAGA	391,212	390,900 (99.92)	195,298	304 (0.08)
L12-6b	GCTACGCT-GTAAGGAG	487,232	486,839 (99.92)	243,224	391 (0.08)
L12-6c	GCTACGCT-ACTGCATA	452,550	452,165 (99.91)	225,891	383 (0.08)
L12-3a	GCTACGCT-AAGGAGTA	538,220	537,775 (99.92)	268,669	437 (0.08)
L12-3b	GCTACGCT-CTAAGCCT	435,300	434,859 (99.90)	217,211	437 (0.10)
L12-3c	CAGAGAGG-TAGATCGC	336,196	335,887 (99.91)	167,790	307 (0.09)
H12-6a	CAGAGAGG-CTCTCTAT	381,304	380,953 (99.91)	190,302	349 (0.09)
H12-6b	CAGAGAGG-TATCCTCT	443,978	443,601 (99.92)	221,614	373 (0.08)
H12-6c	CAGAGAGG-AGAGTAGA	375,458	374,673 (99.79)	187,112	449 (0.12)

L18-1a	CAGAGAGG-GTAAGGAG	509,020	508,165 (99.83)	253,797	571 (0.11)
L18-1b	CAGAGAGG-ACTGCATA	477,902	477,456 (99.91)	238,506	444 (0.09)
L18-1c	CAGAGAGG-AAGGAGTA	598,110	597,048 (99.82)	298,197	654 (0.11)
H18-3a	CAGAGAGG-CTAAGCCT	360,804	360,435 (99.90)	180,035	365 (0.10)
H18-3b	TAGGCATG-TAGATCGC	810	637 (78.64)	312	13 (2.04)
H18-3c	TAGGCATG-CTCTCTAT	413,530	413,154 (99.91)	206,391	372 (0.09)
H18-6a	TAGGCATG-TATCCTCT	378,900	378,548 (99.91)	189,100	348 (0.09)
H18-6b	TAGGCATG-AGAGTAGA	469,864	469,392 (99.90)	234,473	446 (0.10)
H18-6c	TAGGCATG-GTAAGGAG	562,372	561,851 (99.91)	280,692	467 (0.08)
L18-3a	TAGGCATG-ACTGCATA	530,776	530,354 (99.92)	264,966	422 (0.08)
L18-3b	TAGGCATG-AAGGAGTA	659,600	658,995 (99.91)	329,221	553 (0.08)
L18-3c	TAGGCATG-CTAAGCCT	527,752	527,302 (99.91)	263,426	450 (0.09)
H18-1a	CTCTCTAC-TAGATCGC	470,210	469,803 (99.91)	234,701	401 (0.09)
H18-1b	CTCTCTAC-CTCTCTAT	352,674	352,383 (99.92)	176,046	291 (0.08)
H18-1c	CTCTCTAC-TATCCTCT	558,402	557,930 (99.92)	278,729	472 (0.08)
L18-6a	CTCTCTAC-AGAGTAGA	1,108,812	1,107,840 (99.91)	553,439	962 (0.09)
L18-6b	CTCTCTAC-GTAAGGAG	149,350	149,199 (99.90)	74,524	151 (0.10)
L18-6c	CTCTCTAC-ACTGCATA	508	502 (98.82)	248	6 (1.20)

[1] After adapter and quality trimming.

[2] Percentage of the reads remaining after adapter and quality trimming.

[3] Percentage of the trimmed reads that are singletons.

Table S2: Summary of sequences aligned to any of the newly defined OTUs.

Sample	Number of sequences	Number of sequences aligned to any taxa (%)
L6-1a	233,079	224,972 (96.52)
L6-1b	206,654	200,239 (96.90)
L6-1c	222,867	216,582 (97.18)
H6-3a	234,495	225,508 (96.17)
H6-3b	246,354	238,111 (96.65)
H6-3c	203,131	196,827 (96.90)
L6-6a	175,847	171,070 (97.28)
L6-6b	186,586	181,699 (97.38)
L6-6c	206,428	199,807 (96.79)
H6-1a	185,303	179,797 (97.03)
H6-1b	190,821	185,351 (97.13)
H6-1c	172,897	167,522 (96.89)
L6-3a	209,227	201,419 (96.27)
L6-3b	177,165	169,863 (95.88)
L6-3c	239,575	230,603 (96.26)
H6-6a	213,272	204,641 (95.95)
H6-6b	171,816	161,862 (94.21)
H6-6c	226,270	219,962 (97.21)
H12-3a	372,525	359,757 (96.57)
H12-3b	229,830	223,337 (97.17)
H12-3c	234,335	226,636 (96.71)
L12-1a	199,436	193,818 (97.18)
L12-1b	231,452	224,314 (96.92)
L12-1c	200,733	193,784 (96.54)
H12-1a	209,160	203,199 (97.15)
H12-1b	223,185	216,025 (96.79)
H12-1c	209,678	203,797 (97.20)
L12-6a	193,959	187,099 (96.46)
L12-6b	241,742	234,179 (96.87)
L12-6c	224,182	217,494 (97.02)
L12-3a	267,072	260,237 (97.44)
L12-3b	215,877	211,268 (97.86)
L12-3c	166,223	162,130 (97.54)
H12-6a	189,117	182,936 (96.73)
H12-6b	220,290	213,680 (97.00)
H12-6c	185,785	177,992 (95.81)
L18-1a	252,156	244,323 (96.89)
L18-1b	236,919	229,456 (96.85)
L18-1c	296,265	287,235 (96.95)
H18-3a	178,821	172,904 (96.69)
H18-3b	80	52 (65)
H18-3c	205,103	197,131 (96.11)
H18-6a	187,715	180,607 (96.21)
H18-6b	232,648	224,862 (96.65)
H18-6c	278,935	269,764 (96.71)
L18-3a	262,995	254,026 (96.59)
L18-3b	327,129	318,350 (97.32)
L18-3c	261,653	254,576 (97.30)
H18-1a	232,941	226,464 (97.22)
H18-1b	174,826	169,793 (97.12)
H18-1c	276,836	268,880 (97.13)
L18-6a	549,668	532,309 (96.84)
L18-6b	74,063	71,691 (96.80)
L18-6c	213	148 (69.48)

Table S3: Major species (OTUs) for all treatments listed by taxon. Filled cells indicate absence of species in that sample.

Taxa	L6-1a	L6-1b	L6-1c	L6-6a	L6-6b	L6-6c	L6-3a	L6-3b	L6-3c	L12-1a	L12-1b	L12-1c	L12-6a	L12-6b	L12-6c	L12-3a	L12-3b	L12-3c	L18-1a	L18-1b	L18-1c	L18-3a	L18-3b	L18-3c	L18-6a	L18-6b
**Bacteria**	4639	4831	4216	3119	2768	3185	3155	3415	3593	2411	4496	4640	1939	2664	3443	5343	3359	3288	4926	5142	6277	5003	5015	4347	5997	949
**Bacteroidetes**		2142		1860				1949			2354	2330			2874					2298	2944					
**Bacteroidales**																										
**Bacteroidales**											2265			2709	4213						3152					
**Flammeovirgaceae**															2233											
**SB-1**		2251		1724		2175	2321				3929	2091			3489											
**Flavobacteriaceae**	20111	12722	15270	13396	17617	18541	22475	16989	24834	20102	17070	16895	20734	24087	20929	15272	11427	8450	16757	15135	19012	15815	24338	17617	44585	5799
Eudoraea adriatica	21434	16759	20303	12609	16208	14042	15290	12157	18856	13622	12366	12944	14557	15099	16118	25553	26081	19609	13976	10590	12130	8227	16498	15541	29618	4749
***Lutibacter* Sp.**						2216	2373	1746	2582																	
Lutibacter litoralis	4638	3468	3421									4131														
***Lutimonas* Sp.**	2580		2178	1961	2710	2720	3689	2330	3966	2245			2816	3206	2471										6222	1062
***Robiginitalea* Sp.**	7742	3586	4623	4001	9056	7448	11607	7270	11904	12803	7900	4515	25036	28889	17686	46338	48384	39910	33147	26629	33372	32009	47947	42569	110339	13610
***Ulvibacter* Sp.**				2314	2289	2616	2153	2294	2736																	
**Saprospiraceae**	7210	5915	6814	5844	5920	6626	5287	4956	6700	4488	5273	5641	3428	4598	5130	6026	4231	2926	5898	5942	7198	4762	6047	4633	10567	1744
**CFB-26**																										
**Caldilineaceae**																			3587	2847	3799	2798	3998		7066	
**GCA004**	11162	10354	8939	4919	4141	5781	5383	5556	6308	6927	9025	11796	4680	6466	5870	7368	3643	4846	9995	10223	13735	12880	13145	8735	9619	2045
**Ellin6529**																										
**A4b**	2672	3102	2842	2185	2525	3037		1873				3195				3049		2646	3658	3298	4310	3718	4668	3597		
**Stramenopiles**							3635	3318	3620		3671	2081														
***Planktothrix* Sp.**																13258	13413	5770								
**CL500-15**										2148												2872		3191		726
**agg27**		2100						1945	2425							2911	2308	1941	2497							
**Phycisphaerales**	3036	3572	3164	2052	1945		2393	2335	3338	2793	2729	2464			2234	4314	2976	2628	3421	3189	4297	3019	3434			819
**Phycisphaeraceae**				1819	1919		2289		2736	3834	2433								2778							
**Pirellulaceae**													1898			3384	2668	2163	3404	3388	4158	3297	4345		6310	1049
**Rhodobacteraceae**	3459	2127	3585	2859	3410	3551	3390	1955	3622	3878	3185	2675	4344	6404		5471	4751	2944	5329	3948	4214	6971	9477	9672	16688	1878
***Loktanella* Sp.**	3853	2251	4458	3796	5387	4975	4287	2921	4386	5699	3534	2266	5142	6730	2393	3757	4372	2552	3609	2747	3107	2816	7635	4651	13811	1344
***Hydrogenophaga* Sp.**				2738	2808	2903																				
Methylotenera mobilis					2063												2126									
**Desulfobacteraceae**										3694	5299		2808	4410	6389				3428	4689	7403	6131	3515			811
**Desulfobulbaceae**										1820																
**Desulfobulbaceae**		2055																						2620		
**Desulfuromonadaceae**	4185	4650	3866	2325	2280	3387	3795	3017	3984	3053	4134	4400	2035	3369	3340				2692	3254	3719	2653				
**Gammaproteobacteria**	2738	3220	3136	2608	2054	2683	2685	2817	3526	2377	3536	2626	1885	2660	3064	3238			3060	3199	3881	3447	3848	3476	5336	
**Gammaproteobacteria**				1936			2352	2361	2434		4140	2690			2379											
***Glaciecola* Sp.**																										
**OM60**	4080	4431	5886	5499	4631	4531	6326	5867	8698	5586	6773	3709	3836	4349	4737	3355	2156		3170	3138	3798	2729	3826		6031	1032
***Congregibacter* Sp.**																						3824		5870		
**Piscirickettsiaceae**	7709	8904	9141	6846	6287	5879	6506	6892	10044	7961	8407	6191	5581	7711	7722	8654	5295	4023	8378	8190	9810	7693	9594	8475	13784	2051
**Marinicellaceae**	4893	4342	4615	3306	2722	3448	3861	3842	5154	4257	4645	3878	3907	5220	4609	5599	3255	2026	5025	5084	6098	5428	7419	6389	10863	1369
***Marinicella* Sp.**	2471	2200	2714	1863								2367														
**SR1**																2814		2254								
**TM7-1**									2655																	
**Verrucomicrobiaceae**			2675		1972		2635	1741	2674																	
***Luteolibacter* Sp.**			3001	2076									1877													
**KSB4**	4166	4532	3884	2710	2277	2891	2366	1964		4221	4516	3576	2523	3417	4483				2989	3365	4411	3755	4343	3962		868
***B-42* Sp.**																						4789	7282	5266	12010	917
***GBI-58* Sp.**																						7548	5823		26363	879
**Minor <1%**	102194	90725	97851	74705	78710	97172	81166	68353	89828	81719	102634	86683	78073	102191	91688	94533	70823	54154	102599	103161	126410	101842	126153	103965	197100	27990
**SUM**	**122778**	**109514**	**118731**	**96365**	**102989**	**102635**	**120253**	**101510**	**140775**	**113919**	**121680**	**1E+05**	**109026**	**131988**	**125806**	**165704**	**140445**	**107976**	**141724**	**126295**	**160825**	**152184**	**192197**	**150611**	**335209**	**43701**

Taxa	H6-3a	H6-3b	H6-3c	H6-1a	H6-1b	H6-1c	H6-6a	H6-6b	H6-6c	H12-3a	H12-3b	H12-3c	H12-1a	H12-1b	H12-1c	H12-6a	H12-6b	H12-6c	H18-3a	H18-3c	H18-6a	H18-6b	H18-6c	H18-1a	H18-1b	H18-1c
**Bacteria**	4696	5160	4264	3167	2812	2547	4243	2415	4056	6175	4151	3728	2313			4371	4243	4351	3567	5238	4168	4888	7061	3788	2645	5305
**Bacteroidetes**	2512	2621	2246																1831				2842			2892
**Bacteroidales**																										
**Bacteroidales**													2305									2378	3013			2967
**Flammeovirgaceae**																										
**SB-1**		4222				1811			2963		2612	2487														
**Flavobacteriaceae**	19941	17610	16572	14967	18514	13961	17633	16566	22544	42377	22651	22017	21878	23659	21257	9561	11000	10350	12540	12658	10859	13654	16793	18880	14137	19495
Eudoraea adriatica	17040	11306	16201	17393	15143	14403	12200	16270	15407	27743	15330	13389	16959	20922	19165	14520	23396	18190	8076	12397	9805	10975	9353	15304	14007	15178
***Lutibacter* Sp.**					2078	1774	2302		2964																	
Lutibacter litoralis	3096		4345																							
***Lutimonas* Sp.**				2498	3001	2686	2617	2662	3387	5071	2618	2601	3068	3256	3082									2878	2243	3794
***Robiginitalea* Sp.**	6637	6784	4716	6984	6226	4965	7075	11386	10005	21603	11545	11308	26628	35082	28210	27665	39317	28013	17439	20458	18828	25783	24859	42373	34028	37752
***Ulvibacter* Sp.**				2404	3017	2010		1639	2242																	
**Saprospiraceae**	6813	5462	6357	4967	5641	5148	5764	3941	6046	9297	5655	4818	3715	3758	3879	3751	4215	3721	5108	4418	4035	4581	6739	5629	4012	6948
**CFB-26**																				2342						
**Caldilineaceae**																2538	2255		1936		3192		3253	2986	1744	3402
**GCA004**	9856	7470	9318	5411	4417	3661	8232	3427	9428	11444	7788	7890	6230	4762	4364	6040	5825	4178	5621	9246	8274	12288	13101	8150	4548	12136
**Ellin6529**																			1899							
**A4b**	4083		2907	2184		2347			2621							2833	2259	2132	2821	4218	2869	2683	3900			
**Stramenopiles**		5324					4873	2458		3950		2420						2263		1997	1941					
***Planktothrix* Sp.**																13680	11667	6162	1730							
**CL500-15**		2512									2436	2424								2081		2344		3079	2327	2860
**agg27**		2397		1939			2404	2112	2649	3765		2300		2178		1858	2648		1922		1871			2443	2140	
**Phycisphaerales**	2746	3182	2594	2568	2301		3443	2388	3701	4224	2912	3633				2742	3699	2608	3112	3428	2503	2994	3792	3150	2634	3131
**Phycisphaeraceae**		3238			2444		2448	2642	2838	7161	4025	4131		2780				2023	2381	3588	2009			3161	2792	
**Pirellulaceae**				2175			2090						2196		2230	2722	2739		2932		3655	4085	4069	3206	2190	3692
**Rhodobacteraceae**	3539	2891	2690	2845	3365	2984	2619	3373	2741	7170	3946	3967	4612	5536	5368	3333	4287	2163	3398	2478	3265	5689	3496	4009	3334	4598
***Loktanella* Sp.**	3763	2473	2778	4080	4696	4369	2477	4644	3137	8176	4803	4441	5583	6613	6864	2476	3606	3056	1892	2420	2226	2821		3379	2589	
***Hydrogenophaga* Sp.**				2071	3206	3728																				
Methylotenera mobilis										3856				2218												
**Desulfobacteraceae**		5228								4669	4404	5249	3801	2019					1885	3245	3254	6768	10338	2895		5938
**Desulfobulbaceae**		2454								4319	2379	2337											2927			
**Desulfobulbaceae**																						2790	3671			2971
**Desulfuromonadaceae**	3672	3597	4360	2344	2061	2857	3862		4948	5300	3830	4006	2471						2158	2299	1973	2820	3879			2795
**Gammaproteobacteria**	3373	4318	2840	2878	2556	1944	3288	2474	3115	4673	3435	3306			2150	2123	2491	2456	2492	3169	2683	2708	3814		1781	3197
**Gammaproteobacteria**	3119	5926					4545	2090										3483								
***Glaciecola* Sp.**	3664	2618																								
**OM60**	5036	7715	3909	4923	5396	5161	6602	5637	7586	11081	7455	6830	3540	4787	4083	1896	2442	2096	2313	2193	2171	2717	3448	2974	2154	4164
***Congregibacter* Sp.**																										
**Piscirickettsiaceae**	8109	9990	7313	7209	7235	5042	8226	6147	9029	15342	10259	9815	5197	5923	6765	5366	7113	6011	7385	8135	6874	6542	9125	5954	4649	8681
**Marinicellaceae**	4746	4829	4036	3435	3758	3401	4543	2337	4015	6266	5739	5347	3646	3519	4181	2792	3138	2966	3658	3918	4131	5989	7554	4461	2835	6213
***Marinicella* Sp.**	2935		2382	1999	2497																					
**SR1**																2704	2453	2214	1768							
**TM7-1**								1731		3678																
**Verrucomicrobiaceae**				1959	2134	2504	2366	3364																		
***Luteolibacter* Sp.**					2306	2726		1782						2322	2476											
**KSB4**	3547	4188	3719	2305	1911	2577	2515		3822	5579	4337	5576	2562	2331					2179	3158	2073	3817	4661	3194	1702	4864
***B-42* Sp.**																			1812	2251	2632	2468	2966	3123	2321	
***GBI-58* Sp.**																								3433	2437	
**Minor <1%**	102585	104596	93280	77092	78636	74916	88274	60377	90718	136838	91027	92616	86495	84360	89723	69965	74887	69556	69049	81796	75316	93080	115110	78015	56544	105907
**SUM**	**122923**	**133515**	**103547**	**1E+05**	**106715**	**92606**	**116367**	**101485**	**129244**	**222919**	**132310**	**1E+05**	**116704**	**131665**	**114074**	**112971**	**138793**	**108436**	**103855**	**115335**	**105291**	**131782**	**154654**	**148449**	**113249**	**162973**

Figure S2: Relative abundance of bacterial community compositions for 54 sediment samples at species level, including taxonomic identification for all sequences, including both the major (>1% of the total bacterial community) and minor (<1% of the total bacterial community) species. The species legend can be viewed at http://www.cgr.liv.ac.uk:8088/illum/eco_ee57c8f17b78a3e4/

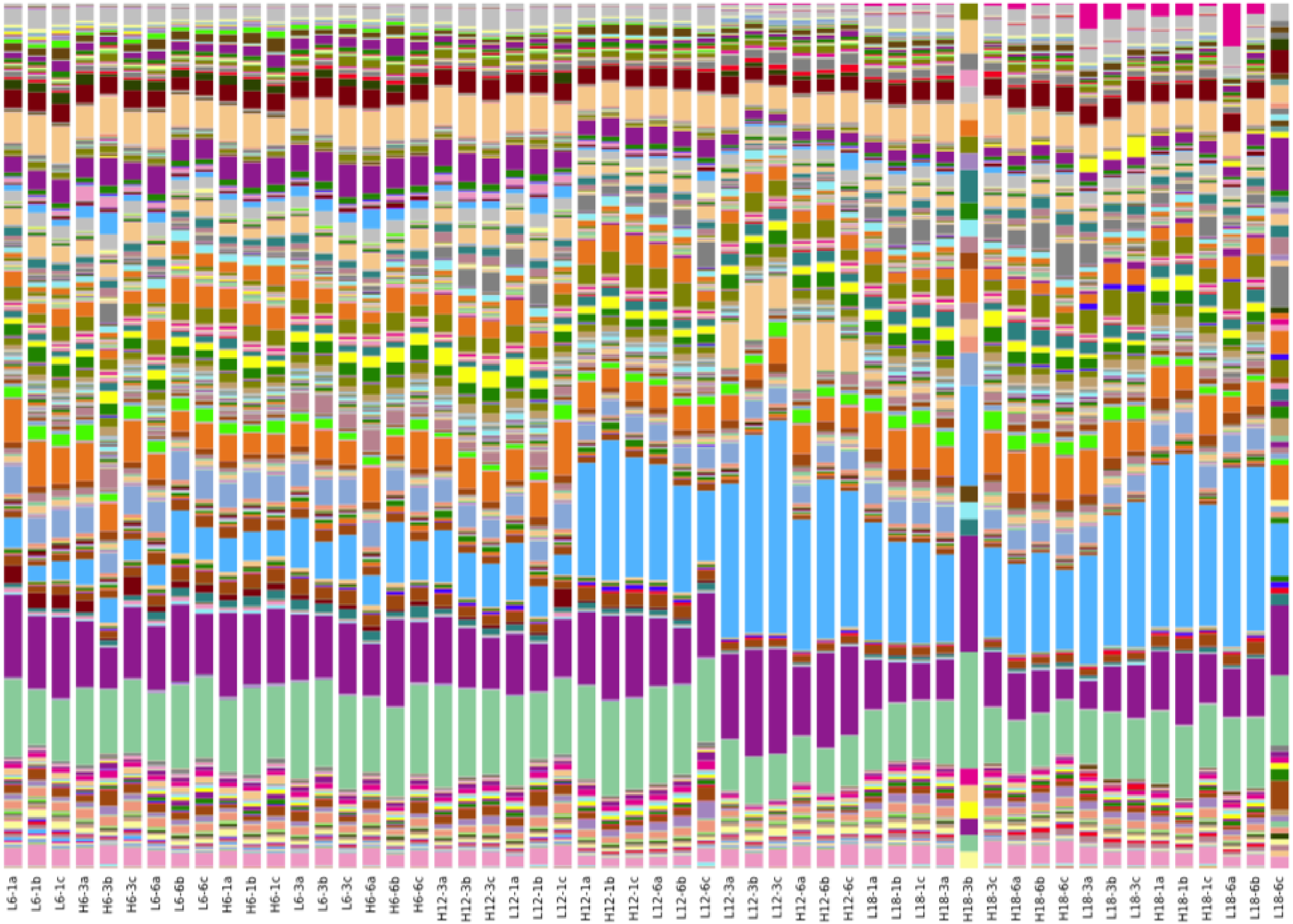

Figure S3: Alpha diversity rarefaction curves, showing (a) observed species (b) phylogenetic diversity and (c) Chao1 diversity estimate for all sediment samples.

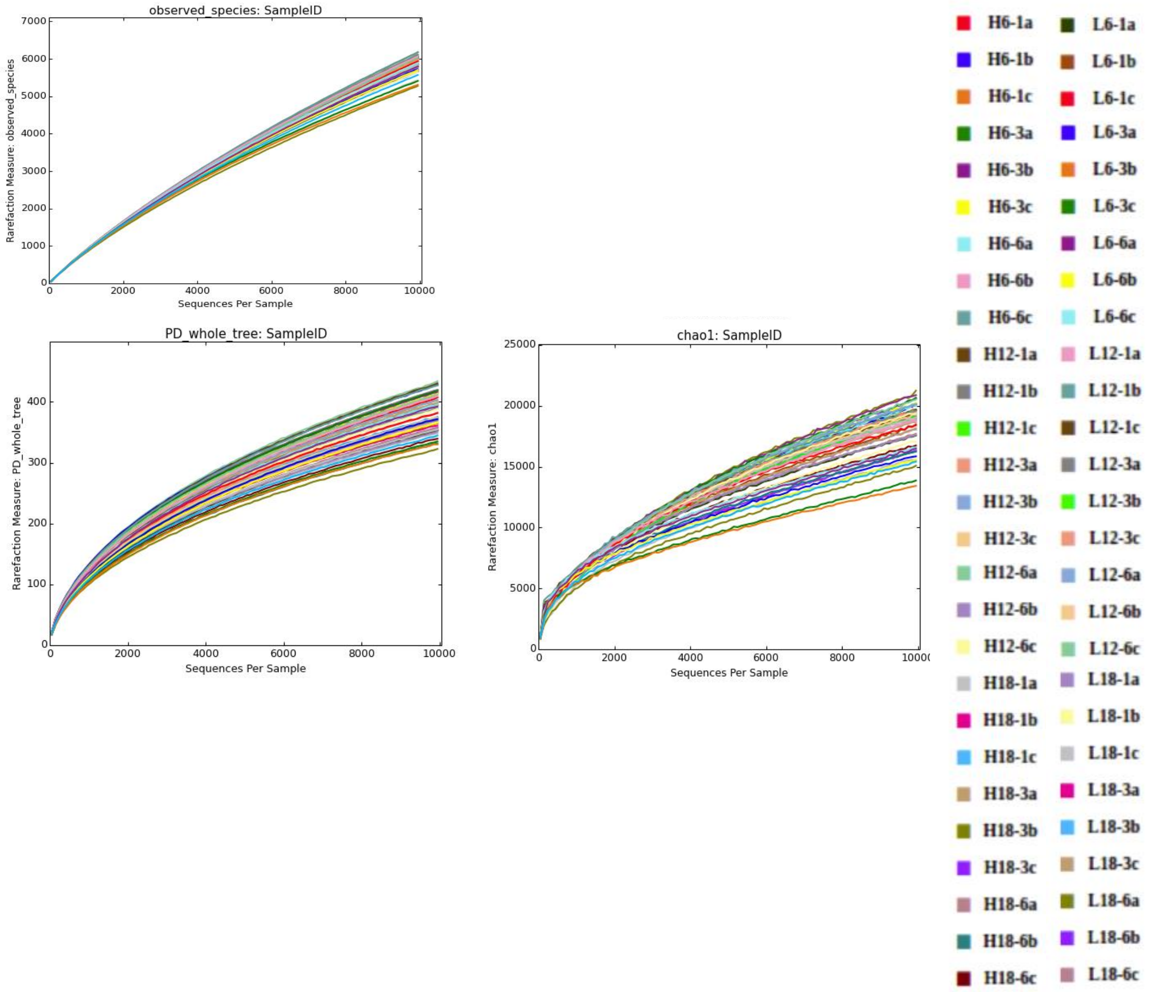

Table S4: Values for observed OTU richness, predicted richness using the Chao1 estimator and phylogenetic distance when sequence sample size was normalized to 9,950 sequences per sample.

Sample	Observed Richness	Chao1	Phylogenetic Distance
L6-1a	6,014	20,142	407.44
L6-1b	6,054	20,125	428.13
L6-1c	5,951	19,688	402.49
H6-3a	6,075	20,664	418.15
H6-3b	6,040	20,877	415.58
H6-3c	5,987	20,234	413.37
L6-6a	5,954	20,012	399.35
L6-6b	5,873	18,913	368.90
L6-6c	5,961	20,120	389.24
H6-1a	6,045	19,347	395.33
H6-1b	5,850	19,320	370.02
H6-1c	5,819	18,988	372.78
L6-3a	5,962	19,400	382.24
L6-3b	6,066	20,151	403.60
L6-3c	5,910	19,307	374.01
H6-6a	6,033	20,510	411.77
H6-6b	5,795	17,614	350.83
H6-6c	5,906	19,348	388.31
H12-3a	5,840	19,047	354.48
H12-3b	5,866	19,600	361.11
H12-3c	5,931	19,640	375.37
L12-1a	5,821	18,627	353.17
L12-1b	6,084	21,245	414.31
L12-1c	5,981	20,595	419.63
H12-1a	5,908	17,708	358.39
H12-1b	5,712	16,235	331.64
H12-1c	5,786	16,739	339.39
L12-6a	5,860	17,566	353.25
L12-6b	5,904	18,402	364.87
L12-6c	5,937	18,461	381.66
L12-3a	5,728	15,848	371.77
L12-3b	5,310	13,424	330.09
L12-3c	5,410	13,865	334.96
H12-6a	5,799	16,494	392.31
H12-6b	5,708	15,586	365.63
H12-6c	5,886	16,586	395.45
L18-1a	6,070	18,729	409.47
L18-1b	6,179	19,564	427.13
L18-1c	6,181	19,674	430.54
H18-3a	6,114	19,139	419.28
H18-3b	N/A	N/A	N/A
H18-3c	6,070	18,794	419.21
H18-6a	5,995	18,833	407.53
H18-6b	5,998	19,110	409.18
H18-6c	6,125	20,005	433.92
L18-3a	5,775	18,145	394.30
L18-3b	5,656	17,055	364.73
L18-3c	5,427	15,518	348.38
H18-1a	5,678	16,290	353.99
H18-1b	5,570	15,417	344.71
H18-1c	5,984	18,045	398.59

L18-6a	5,277	15,079	322.62
L18-6b	5,657	16,337	357.51
L18-6c	N/A	N/A	N/A

Figure S4 (a): Non-metric multidimensional scaling plots of bacterial community composition with labels coloured according to CO_2 level: 380 ppmv (blue) and 1000 ppmv (red).

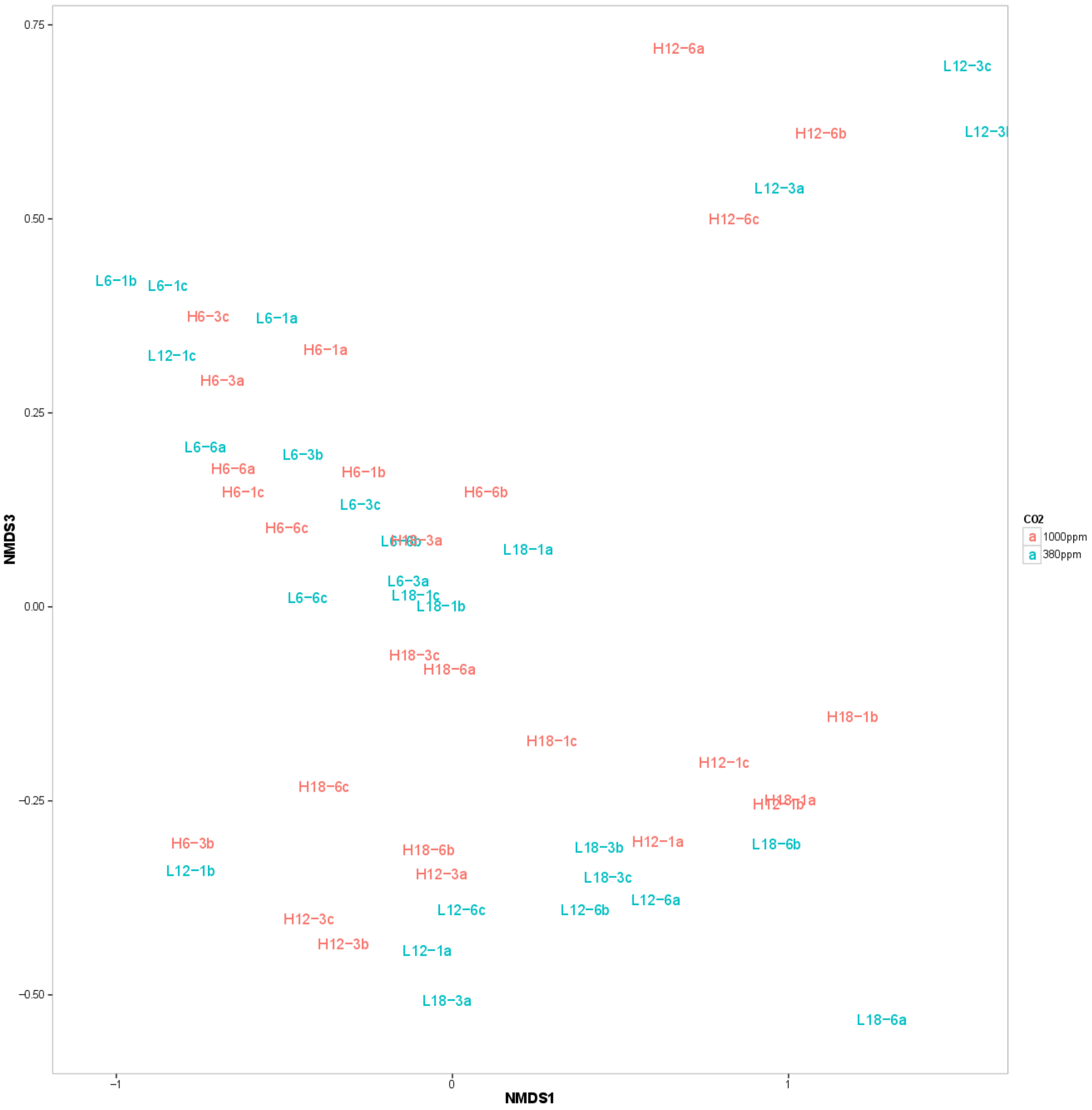

Figure S4 (b): Non-metric multidimensional scaling plots of bacterial community composition with labels coloured according to temperature fluctuation: 1 °C (red), 3 °C (green) and 6 °C (blue).

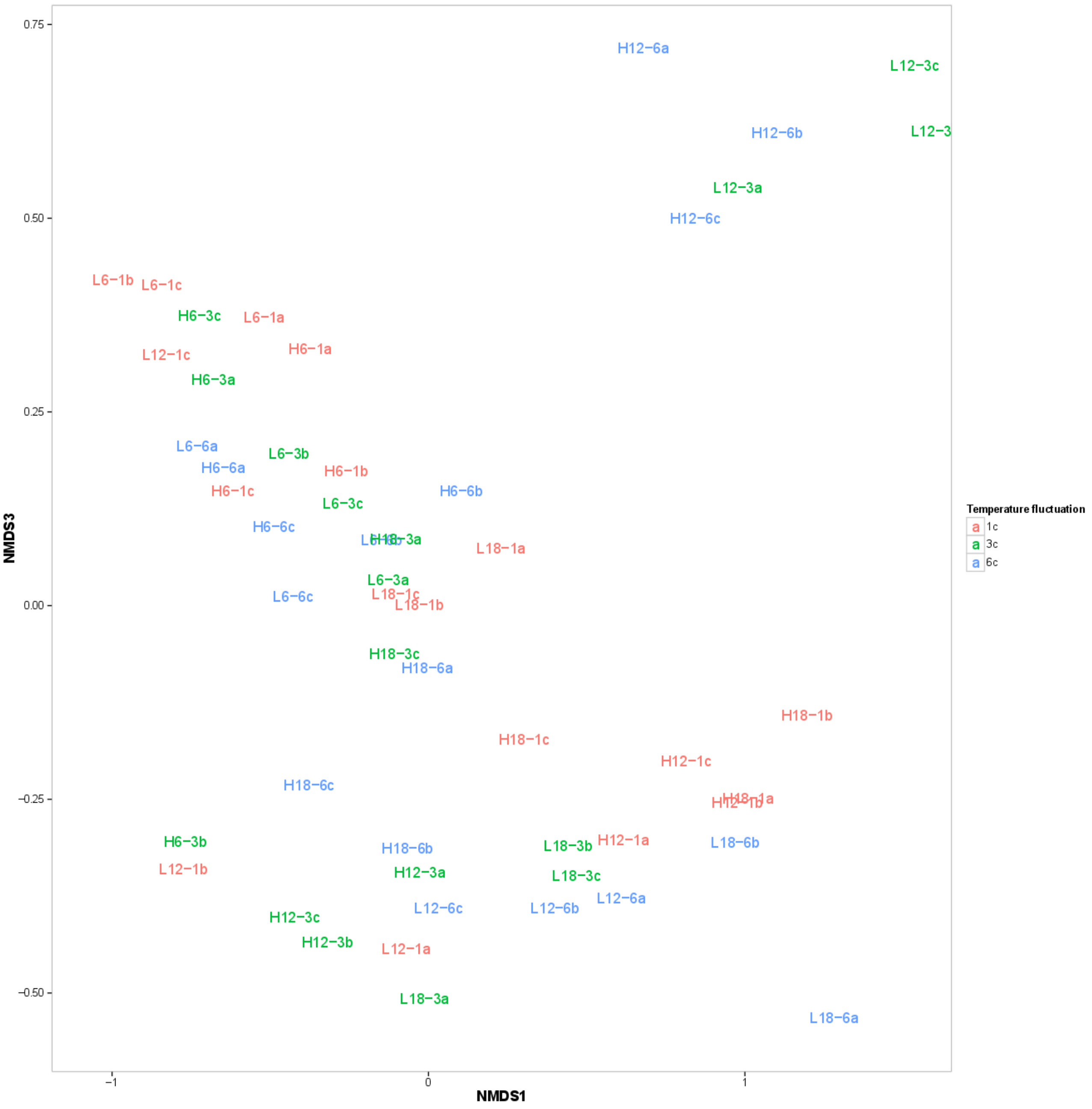

Detailed methodology

16S rRNA gene amplification and sequencing

Amplicon generation using the primers 5'ACACTCTTTCCCTACACGACGCTCTTCCGATCTNNNNNGTGCCAGCMGCCGCGGTAA3' and 5'GTGACTGGAGTTCAGACGTGTGCTCTTCCGATCTGGACTACHVGGGTWTCTAAT3'

(targeting the 16S rRNA gene) was performed for each of the 54 environmental DNA samples. These were amplified by 10 cycles of PCR using the Kapa enzyme. The samples were then cleaned using a 1:1 ratio of AMPure XP beads on a Tecan Evo150 robot. The samples then underwent a further round of PCR, during which the barcode indexes and Illumina adapters were incorporated into the final PCR products. Fifteen cycles were performed using the Kapa enzyme and the samples cleaned again using a 1:1 ratio of AMPure XP beads on the Tecan robot.

The DNA concentrations were recorded using a Qubit fluorometer (ThermoFisher) and scanned on the Fragment Analyser (Advanced Analytical) and this data was used to pool the samples. The pooled sample was then size-selected at 350-650 base pairs (bp) using the Pippin Prep (Sage). The size-selected sample was then cleaned using Ampure XP beads and the quality and quantity of the pool was assessed again using Qubit and the Fragment Analyser and subsequently by qPCR using the Illumina Library Quantification Kit from Kapa on a Roche Light Cycler LC48011 according to manufacturer's instructions. The template DNA was denatured according to the protocol described in the

Illumina cBot user guide and loaded at a concentration of 8.5 pM. To increase sample diversity 15% PhiX was spiked into the sample. The sequencing was carried out on one lane of an Illumina MiSeq at 2x250 base pair (bp) paired-end sequencing with v2 chemistry. Sequences are published in the European Nucleotide Archive (ENA) under the study accession number PRJEB13670 and sample accession numbers ERS1124371-ERS1124422.

Initial processing and quality assessment of the sequence data

Quality control and processing of the pooled amplicon sample (including indexes and the addition of Illumina adaptors) was followed by paired-end sequencing (2x250 bp) using the Illumina MiSeq platform at the University of Liverpool Centre for Genomic Research NERC Biomolecular Analysis Facility. Fragmented PhiX phage was added to the sequence library in order to increase the sequence complexity. PhiX sequences are not indexed, so should not be associated with indexed reads after de-multiplexing. However, a small proportion of PhiX reads can become associated with an index (probably due to 'cross-talk' between adjacent clusters on the sequencing flowcell). To remove these sequences, each sample was compared with the complete PhiX sequence (GenBank gi9626372) using BLASTn (Altschul et al., 1990). Sequences matching PhiX (E-value < 10^{-5}) were filtered out of the dataset. A custom script was used to exclude sequences with lengths outside of the expected range (< 200 bp or > 350 bp), which are likely to represent errors.

Initial processing and quality assessment of the sequence data was performed using an in-house pipeline developed by Dr Richard Gregory at the University of Liverpool Centre for Genomic Research. Briefly, basecalling and de-multiplexing of indexed reads was performed by CASAVA version 1.8.2 (Illumina) to produce 70 samples from the single flowcell, in fastq format. The raw fastq files were trimmed to remove Illumina adapter sequences using Cutadapt version 1.2.1 (Martin, 2011). The option "-O 3" was set, so the 3' end of any reads which matched the adapter sequence over at least 3 bp was trimmed off. The reads were further trimmed to remove low quality bases, using Sickle version 1.200 with a minimum window quality score of 20. After trimming, reads shorter than 10 bp were removed. If both reads from a pair passed this filter, each was included in the R1 (forward reads) or R2 (reverse reads) file. If only one of a read pair passed this filter, it was included in the R0 (unpaired reads) file. FLASh version 1.2.8 (Magoc and Salzberg, 2011) was used to assemble each pair of reads into a single sequence representing the complete amplicon. Read pair sequences were assembled into single sequences spanning the amplicon and filtered for sequences derived from PhiX phage (routinely added to the sequence libraries).

Grouping sequences into operational taxonomic units

A metadata file was created to describe each sample. These two files were used for metagenomic analysis using Qiime, version 1.8.0 (Caporaso et al., 2010b). To correct incorrect clustering resulting in overestimation of the number of OTUs, an error calculation was run by clustering sequences at 99% identify and generating a consensus sequence for the cluster. Additionally chimera detection used a dataset of 16 rRNA genes

as potential 'parent' sequences in addition to using the most common sequences in the dataset. Both filters were run as a single step using the USEARCH7 "uchime_ref" option using the Greengenes database of ribosomal RNA sequences, version 12.8 (McDonald et al., 2012) as a comparison set of reference sequences for chimera detection.

The first chimera detection step used a database of 16S rRNA genes as potential "parent" sequences from which chimeras may be derived. The second used the most common sequences in the data itself as potential "parent" sequences (hence *de novo*). There were 582,651 sequence clusters that passed the chimera filters. Errors are expected to be rare compared to corrected sequences, therefore, clusters containing few sequences are more likely to be the result of errors compared to clusters containing many sequences.

Post-processing of the Illumina sequence reads included quality control, transfer of *sff data to sequence (*fna) and quality (*qual) files and clustering of reads into operational taxonomic units (OTUs) at 99% sequence identity. A minimum cluster size was set to remove clusters containing fewer than four sequences. OTU-picking was done following the Qiime script "pick_otus.py" using USEARCH7 (Edgar et al., 2011) to cluster sequences, removing chimeras and define OTU abundance. Taxonomic assignment of each OTU was achieved using the Qiime script "assign_taxonomy.py" using the RDP classifier (Wang et al., 2007) to match a representative sequence from the OTU to a sequence from the Greengenes database, version 12.8 (McDonald et al., 2012), clustering at 97% identify with associated taxonomic information.

Alpha diversity and rarefaction statistical analysis

OTU tables were repeatedly sub-sampled using the Qiime script "multiple_rarefactions.py". The Chao1 index was calculated using the following formula:

$$S_1 = S_{obs} + \frac{F_1^2}{2F_2}$$

Where S_{obs} was the observed number of species within a sample, F1 was the number of singleton species (species which occur once within a sample) and F2 was the number of doubletons (species which occur twice within a sample) (Chao, 1984; Colwell and Coddington, 1994).

Beta diversity analysis including UPGMA trees and 2D-PCoA plots

A phylogeny was achieved by generating a sequence alignment of the representative sequences of each OTU via the Qiime script "align_seqs.py" using Pynast (Caporaso et al., 2010a) to align the sequences to a set of pre-aligned 16S rRNA sequences (bundled within Qiime). A minimum length of 150 bp and identify of 75% were the criteria for inclusion in the alignment. The alignment was filtered, using the Qiime script "filter_alignment.py" to remove uninformative sites (based on a file of phylogenetically informative and uninformative positions in the 16S rRNA gene pre-alignment) and sites with gaps in >95% of sequences. Finally the filtered alignment was used to build a phylogeny via the Qiime script "make_phylogeny.py" using the FastTree algorithm (Price et al., 2010).

All datasets were sub-sampled (rarefied) using the Qiime script "multiple_rarefaction_even_depth.py". These OTU tables were used to calculate weighted and unweighted pair-wise UniFrac matrices using the Qiime script "beta_diversity.py". UniFrac matrices were then used to generate Unweighted Pair-Group Method with Arithmetic Mean (UPMGA) trees and 2D principle coordinates plots (PCoA) using the following Qiime scripts "upgma_cluster.py", "consensus_tree.py", "tree_compare.py", "make_bootstrapped_tree.py", "principal_coordinates.py" and "make_2d_plots".

References

Altschul, S.F., Gish, W., Miller, W., Myers, E.W., and Lipman, D.J. (1990). Basic Local Alignment Search Tool. *Journal of Molecular Biology* 215(3), 403-410.

Caporaso, J.G., Bittinger, K., Bushman, F.D., DeSantis, T.Z., Andersen, G.L., and Knight, R. (2010a). PyNAST: a flexible tool for aligning sequences to a template alignment. *Bioinformatics* 26(2), 266-267.

Caporaso, J.G., Kuczynski, J., Stombaugh, J., Bittinger, K., Bushman, F.D., Costello, E.K., et al. (2010b). QIIME allows analysis of high-throughput community sequencing data. *Nature Methods* 7(5), 335-336.

Chao, A. (1984). Nonparametric-Estimation of the Number of Classes in a Population. *Scandinavian Journal of Statistics* 11(4), 265-270.

Colwell, R.K., and Coddington, J.A. (1994). Estimating Terrestrial Biodiversity through Extrapolation. *Philosophical Transactions of the Royal Society of London Series B-Biological Sciences* 345(1311), 101-118.

Edgar, R.C., Haas, B.J., Clemente, J.C., Quince, C., and Knight, R. (2011). UCHIME improves sensitivity and speed of chimera detection. *Bioinformatics* 27(16), 2194-2200.

Magoc, T., and Salzberg, S.L. (2011). FLASH: fast length adjustment of short reads to improve genome assemblies. *Bioinformatics* 27(21), 2957-2963.

Martin, M. (2011). Cutadapt removes adapter sequences from high-throughput sequencing reads. *EMBnet.journal* 17(1), 10-12. doi: http://dx.doi.org/10.14806/ej.17.1.200.

McDonald, D., Price, M.N., Goodrich, J., Nawrocki, E.P., DeSantis, T.Z., Probst, A., et al. (2012). An improved Greengenes taxonomy with explicit ranks for ecological and evolutionary analyses of bacteria and archaea. *Isme Journal* 6(3), 610-618.

Price, M.N., Dehal, P.S., and Arkin, A.P. (2010). FastTree 2-Approximately Maximum-Likelihood Trees for Large Alignments. *Plos One* 5(3).

Wang, Q., Garrity, G.M., Tiedje, J.M., and Cole, J.R. (2007). Naive Bayesian classifier for rapid assignment of rRNA sequences into the new bacterial taxonomy. *Applied and Environmental Microbiology* 73(16), 5261-5267.
